# -196 to -174del, rs4696480, rs3804099 polymorphisms of Toll-like receptor 2 gene impact the susceptibility of cancers: evidence from 37053 subjects

**DOI:** 10.1042/BSR20191698

**Published:** 2019-12-06

**Authors:** Sheng-Lin Gao, Yi-Ding Chen, Chuang Yue, Jiasheng Chen, Li-Feng Zhang, Si-Min Wang, Li Zuo

**Affiliations:** 1 Department of Urology, The Affiliated Changzhou No. 2 People’s Hospital of Nanjing Medical University, Changzhou, Jiangsu, China; 2 The First Clinical College of Anhui Medical University, Hefei, Anhui, China; 3 Department of Ophthalmology, Changzhou Third People’s Hospital, Changzhou, Jiangsu, China

**Keywords:** Cancer risk, Meta-analysis, TLR2, Toll-like receptor 2

## Abstract

Relationship between Toll-like receptor-2 (*TLR2*) and cancer risk has been illustrated in some studies, but their conclusions are inconsistent. Therefore, we designed this meta-analysis to explore a more accurate conclusion of whether *TLR2* affects cancer risks. Articles were retrieved from various literature databases according to the criteria. We used STATA to calculate the odds ratio (OR) and 95% confidence interval (95% CI) to evaluate the relationship between certain polymorphism of *TLR2* and cancer risk. Finally, 47 case–control studies met the criteria, comprising 15851 cases and 21182 controls. In the overall analysis, people are more likely to get cancer because of -196 to -174del in *TLR2* in all five genetic models, B vs. A (OR = 1.468, 95% Cl = 1.129–1.91, *P*=0.005); BB vs. AA (OR = 1.716, 95% Cl = 1.178–2.5, *P*=0.005); BA vs. AA (OR = 1.408, 95% Cl = 1.092–1.816, *P*=0.008); BB+BA vs. AA (OR = 1.449, 95% Cl = 1.107–1.897, *P*=0.007); BB vs. BA+AA (OR = 1.517, 95% Cl = 1.092–2.107, *P*=0.013). Meanwhile, rs4696480 could significantly increase the risk of cancer in Caucasians, furthermore, rs3804099 significantly decreased cancer risk in overall analysis, but more subjects are necessary to confirm the results. All in all, this meta-analysis revealed that not only -196 to -174del increased the risk of among overall cancers, Caucasians are more likely to get cancer because of rs4696480, while rs3804099 polymorphism could reduce the risk of cancer in some genetic models. There is no direct evidence showing that rs5743708, rs3804100 and rs1898830 are related to cancer.

## Introduction

Cancer prevalence increases rapidly and becomes a major threat to human health in today’s world. As we all know, genes are inextricably linked to the development of cancer. In many cancer studies, such as gastric cancer [1], colorectal cancer, breast cancer [2], cervical cancer [3], Toll-like receptor (TLR)-2 (*TLR2*) has been determined as a pathogenic factor involved in tumorigenesis. The *TLR2* gene located on human chromosome 4q32, includes one coding exon and two non-coding exons [4]. TLRs are mainly expressed in immune-related cells and immune-related epithelial cells, their role in tissue resistance to microbes is achieved by identifying conserved bacterial molecules [5]. Therefore some researchers believe that *TLR2* play a significant role in the innate immune response through releasing pro-inflammatory cytokines [6].

-196 to -174del is a 22-bp deletion in *TLR2* gene, which has been shown to cause a decrease in the transcriptional activity of the *TLR2* gene [7]. However, in the past few years, there are inconsistent conclusions about the relationship between -196 to -174del and cancer risk. One paper noted that -196 to -174del in association with *Helicobacter pylori* significantly increased the risk of gastric cancer in patients [1]. But Hishida et al. [8] suggested that -196 to -174del had no relationship with gastric cancer. About reproductive tumors, some literatures suggested that -196 to -174del is not associated with breast cancer [9] and cervical cancer [3], but on the contrary, Theodoropoulos et al. [10] think that -196 to -174del may produce a significant increase in the risk of breast cancer. Mandal et al. [11] revealed that -196 to -174del polymorphism in *TLR2* gene seems to be associated with the upgraded prostate cancer risk, while Singh et al. [12] drew out that -196 to -174del showed a three- to five-folds risk of bladder cancer comparison with people without this mutation.

For rs3804099 (c.597T>C) and rs3804100 (c.1350T>C), Etokebe et al. [13] and Semlali et al. [14] found no association between these two SNPs and breast cancer; Tongtawee et al. [15] demonstrated that rs3804099 and rs3804100 had no relationship with gastric cancer. However, the study of Xie et al. [16] found that the risk of hepatocellular carcinoma in *TLR2* rs3804099 and rs3804100 carriers was reduced. For rs4696480 (g.6686T>A), de Barros Gallo et al. [17] thought that rs4696480 was associated with oral cancer in Caucasians, but Semlali et al. [18] found no difference in rs4696480 expression between the breast cancer patients and the controls in Asians.

Therefore, considering the limitations of individual study sample sizes and the contradictions of their conclusions, we designed this meta-analysis to study the relationship between *TLR2* polymorphisms. (rs3804099, rs3804100, rs4696480, rs5743708 (c.2258G> A), rs1898830 (g.8013A> G) and -196 to -174del) and cancer risk.

## Materials and methods

### Database searching

Up to October 2019, PubMed, Embase, Google Scholar, Web of Science, Wanfang database and CNKI database were used by two investigators for article identification. We used the following strategy for the searching of relevant citations: (TLR2 OR (Toll-like receptors-2) OR CD282) AND (cancer OR tumor OR carcinoma OR neoplasms OR malignancy) AND (polymorphism OR mutation OR variant OR SNP OR genotype). Since the present study is a meta-analysis, no institutional review board approval and patient consent were required.

### Inclusion and exclusion criteria

Articles included in our research must meet the following conditions: (1) study the relationship between cancer risk and *TLR2* polymorphism; (2) provide sufficient data for extraction and calculation; (3) subjects are human patients; (4) the case–control study included control group and cancer patients case group. When duplicate data appeared in different publications, only the latest publication data were used. If the study did not meet the above criteria, it was excluded.

### Data extraction and quality assessment

We extracted data from these articles, such as cancer type, first author, ethnicity, source of control, publication year, number of cases and controls, etc. Any differences were resolved through group discussions until all consensus was reached. We used Newcastle–Ottawa Scale (NOS) to evaluate the quality of the article (http://www.ohri.ca/programs/clinical_epidemiology/oxford.asp). We carefully recorded seven aspects including ‘adequacy of case definition’, ‘representativeness of the cases’, ‘selection of controls’, ‘definition of controls’, ‘comparability cases/controls’, ‘ascertainment of exposure’ and ‘ascertainment of exposure’ to evaluate.

### Statistical analysis

The STATA software was used for meta-statistical analysis. The relationship between the *TLR2* rs3804099, rs3804100, rs4696480, rs5743708, rs1898830, -196 to -174del and cancer risk was assessed using pooled odds ratios (ORs) with 95% confidence intervals (95% CIs) under dominant, recessive, homozygous codominance, heterozygous codominance, and allelic control genetic models. Heterogeneity was estimated using Q test and *I^2^* statistics [19]. When heterogeneity existed (*P*<0.1), random-effects model was applied, otherwise, fixed-effect model was used [20]. The Hardy–Weinberg equilibrium (HWE) of the control group was calculated using the chi-square test. In addition, we performed a stratified analysis based on cancer type, race, source of control and quality score. The sensitivity analysis was used to evaluate the stability of the overall analysis and the publication bias was evaluated by Egger’s test and Begg’s funnel plot [21].

### False-positive report probability analysis and trial sequential analysis

We also used the false-positive report probability (FPRP) to evaluate the results; 0.2 was set as thePRP threshold and assigned a prior probability of 0.25 to detect the OR of 0.67/1.50 (protective/risk effects). The significant result with the FPRP values less than 0.2 were considered a worthy finding [22,23]. Trial sequential analysis (TSA) was conducted with the guideline of a former publication [24,25]. We set a significance of 5% for type I error, as well as a 30% significance of type II error, to calculate the required sample size, and built the TSA monitoring boundaries.

### *In silico* analysis

For evaluating the linkage disequilibrium (LD) between different polymorphisms, we downloaded the dataset including the polymorphisms information of *TLR2* gene from the 1000 Genomes Project, which contained the distribution of gene polymorphisms among CHB (Han Chinese in Beijing, China), CHS (southern Han Chinese, China), CEU (Utah residents with Northern and Western European ancestry from the CEPH collection), JPT (Japanese in Tokyo, Japan) and YRI (Yoruba in Ibadan, Nigeria), ESN (Esan in Nigeria) patients, and we used Haplpoview software to visualize the association between different polymorphisms, the relationship between them were assessed by r^2^ statistics. We also performed the expression quantitative trait loci (eQTL) analysis using GTEx portal website (http://www.gtexportal.org/home/) to predict potential associations between the SNPs and gene expression levels [26,27].

## Results

### Search results

**Figure 1:**
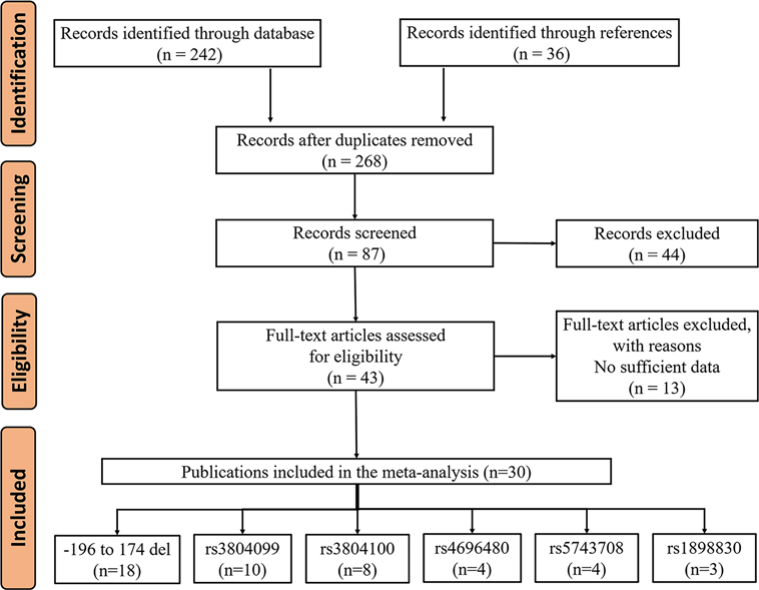
Flowchart of enrolled studies selection procedure

We used online databases to find 242 articles, and found another 36 articles by reviewing the references. After removing the duplicates, we found a total of 268 records in the database. We first screened the duplicate articles and then screened 43 of the high-quality articles on the NOS (Supplementary Table S1). Of the 43 articles selected, 13 were rejected for insufficient data. At last, 30 articles met the criteria, including 47 case–control studies. The flowchart of our study selection is shown in Figure 1. This meta-analysis collected individuals with different genetic backgrounds (e.g. Asians, Africans and Caucasians). The detailed characteristics of these publications are provided in Table 1.

**Table 1:** Characteristics of the enrolled studies on *TLR2* polymorphism and cancer

First author	Year	Ethnicity	Genotyping method	Source of control	Cancer type	Cases						Control						
						AA	BA	BB	Total	A%	B%	AA	BA	BB	Total	A%	B%	HWE
**(-196 to -174del)**																		
Tahara et al.	2007	Asian	AS-PCR	PB	Gastric cancer	126	112	51	289	63.0%	37.0%	73	65	8	146	72.3%	27.7%	Y
Pandey et al.	2009	Asian	PCR	PB	Cervical cancer	102	43	5	150	82.3%	17.7%	114	35	1	150	87.7%	12.3%	Y
Hishida et al.	2010	Asian	PCR	HB	Gastric cancer	243	267	73	583	64.6%	35.4%	722	730	184	1636	66.4%	33.6%	Y
Srivastava et al.	2010	Asian	PCR-RFLP	PB	Gallbladder cancer	132	94	6	232	77.2%	22.8%	163	87	4	254	81.3%	18.7%	N
Zeng et al.	2011a	Asian	DHPLC	HB	Gastric cancer	119	110	19	248	70.2%	29.8%	187	246	63	496	62.5%	37.5%	Y
Nischalk et al.	2011	Caucasian	PCR	PB	Hepatocellular carcinoma	115	63	11	189	77.5%	22.5%	248	92	7	347	84.7%	15.3%	Y
Oliveira et al.	2012	Caucasian	PCR-RFLP	PB	Gastric cancer	116	50	8	174	81.0%	19.0%	189	34	2	225	91.6%	8.4%	Y
Mandal et al.	2012	Asian	PCR	PB	Prostate cancer	135	54	6	195	83.1%	16.9%	193	52	5	250	87.6%	12.4%	Y
Theodoropoulos et al.	2012	Caucasian	PCR	PB	Breast cancer	120	113	28	261	67.6%	32.4%	432	46	2	480	94.8%	5.2%	Y
Singh et al.	2013	Asian	PCR	PB	Bladder cancer	110	79	11	200	74.8%	25.3%	119	73	8	200	77.8%	22.3%	Y
Bi et al.	2014	Asian	PCR	PB	Cervical cancer	40	47	15	102	62.3%	37.7%	36	50	14	100	61.0%	39.0%	Y
Castano-Rodriguez et al.	2014	Asian	MassARRAY	HB	Gastric cancer	7	44	35	86	33.7%	66.3%	19	95	106	220	30.2%	69.8%	Y
Zidi et al.	2014	African	PCR	HB	Cervical cancer	89	20	13	122	81.1%	18.9%	196	37	27	260	82.5%	17.5%	N
Devi et al.	2015	Asian	PCR	PB	Breast cancer	251	191	20	462	75.0%	25.0%	491	246	33	770	79.7%	20.3%	Y
Proenca et al.	2015	African	PCR	PB	Colorectal cancer	144	39	5	188	87.0%	13.0%	200	36	4	240	90.8%	9.2%	Y
Zidi et al.	2015	African	PCR	PB	Cervical cancer	93	26	11	130	81.5%	18.5%	196	37	27	260	82.5%	17.5%	N
AL-Harras et al.	2016	African	PCR-RFLP	PB	Breast cancer	44	22	6	72	76.4%	23.6%	61	33	6	100	77.5%	22.5%	Y
Huang et al.	2018	Asian	PCR	PB	Gastric cancer	105	124	31	260	64.2%	35.8%	132	113	15	260	72.5%	27.5%	Y
**rs3804099**																		
Etokebe et al.	2009	Caucasian	TaqMan	PB	Breast cancer	29	44	16	89	57.3%	42.7%	26	48	15	89	56.2%	43.8%	Y
Slattery et al.	2012	Caucasian	GoldenGate	PB	Colon cancer	1255		300	1555	-	-	1531	425		1956	-	-	-
Xie et al.	2012	Asian	SNaPshot	HB	Hepatocellular carcinoma	19	71	121	211	25.8%	74.2%	15	117	100	232	31.7%	68.3%	N
Miedema et al.	2012	Caucasian	AS-PCR	HB	Lymphoblastic leukemia	51	94	37	182	53.8%	46.2%	48	102	28	178	55.6%	44.4%	N
Slattery et al.	2012	Caucasian	GoldenGate	PB	Rectal cancer	238	372	144	754	56.2%	43.8%	299	477	183	959	56.0%	44.0%	Y
Zeljic et al.	2013	Caucasian	TaqMan	PB	Oral cancer	29	39	25	93	52.2%	47.8%	37	67	0	104	67.8%	32.2%	N
Semlali et al.	2017	Asian	TaqMan	PB	Breast cancer	35	58	32	125	51.2%	48.8%	33	71	42	146	46.9%	53.1%	Y
Semlali et al.	2018	Asian	TaqMan	PB	Colon cancer	42	50	19	111	60.4%	39.6%	28	47	27	102	50.5%	49.5%	Y
Tongtawee et al.	2018	Asian	TaqMan	HB	Gastric cancer	62	13	13	88	77.8%	22.2%	194	56	62	312	71.2%	28.8%	N
Zeng et al.	2011b	Asian	PCR-RFLP	HB	Gastric cancer	132	99	17	248	73.2%	26.8%	216	231	49	496	66.8%	33.2%	Y
**rs3804100**																		
Purdu et al.	2008	Caucasian	TaqMan	PB	Non-Hodgkin lymphoma	1658	272	12	1942	92.4%	7.6%	1556	233	9	1798	93.0%	7.0%	Y
Etokebe et al.	2009	Caucasian	TaqMan	PB	Breast cancer	76	13	0	89	92.7%	7.3%	84	11	0	95	94.2%	5.8%	Y
Xie et al.	2012	Asian	SNaPshot	HB	Hepatocellular carcinoma	14	67	130	211	22.5%	77.5%	11	110	111	232	28.4%	71.6%	N
Miedema et al.	2012	Caucasian	AS-PCR	HB	Lymphoblastic leukemia	170	18	1	189	94.7%	5.3%	165	18	0	183	95.1%	4.9%	Y
Castano-Rodriguez et al.	2014	Asian	MassARRAY	HB	Gastric cancer	47	34	4	85	75.3%	24.7%	122	76	14	212	75.5%	24.5%	Y
Semlali et al.	2017	Asian	TaqMan	PB	Breast cancer	99	24	1	124	89.5%	10.5%	115	27	4	146	88.0%	12.0%	Y
Semlali et al.	2018	Asian	TaqMan	PB	Colon cancer	99	13	2	114	92.5%	7.5%	82	19	2	103	88.8%	11.2%	Y
Tongtawee et al.	2018	Asian	TaqMan	HB	Gastric cancer	66	22	0	88	87.5%	12.5%	230	70	12	312	84.9%	15.1%	N
**rs4696480**																		
Miedema et al.	2012	Caucasian	AS-PCR	HB	Hepatocellular carcinoma	42	99	44	185	49.5%	50.5%	60	83	38	181	56.1%	43.9%	Y
Gallo et al.	2017	Caucasian	TaqMan	PB	Oral cancer	12	39	24	75	42.0%	58.0%	31	34	24	89	53.9%	46.1%	N
Semlali et al.	2017	Asian	TaqMan	PB	Breast cancer	46	51	29	126	56.7%	43.3%	50	63	25	138	59.1%	40.9%	Y
Semlali et al.	2018	Asian	TaqMan	PB	Colon cancer	30	49	27	106	51.4%	48.6%	26	41	25	92	50.5%	49.5%	Y
**rs5743708**																		
Nischalk et al.	2011	Caucasian	PCR	PB	Hepatocellular carcinoma	174	15	0	189	96.0%	4.0%	319	28	0	347	96.0%	4.0%	Y
Slattery et al.	2012	Caucasian	GoldenGate	PB	Rectal cancer	727	27		754	-	-	913	46		959	-	-	
Slattery et al.	2012	Caucasian	GoldenGate	PB	Colon cancer	1467	88		1555	-	-	1864	92		1956	-	-	
Kina et al.	2018	Caucasian	PCR	PB	Glioma	32	18	70	120	34.2%	65.8%	184	35	6	225	89.6%	10.4%	N
**rs1898830**																		
Xie et al.	2012	Asian	SNPshot	HB	Hepatocellular carcinoma	47	92	72	211	44.1%	55.9%	34	118	80	232	40.1%	59.9%	Y
Slattery et al.	2012	Caucasian	GoldenGate	PB	Rectal cancer	305	363	86	754	64.5%	35.5%	410	437	111	958	65.6%	34.4%	Y
Slattery et al.	2012	Caucasian	GoldenGate	PB	Colon cancer	705	674	176	1555	67.0%	33.0%	896	833	227	1956	67.1%	32.9%	Y

Abbreviations: H-B, hospital based; P-B, population based. *P*>0.05 means conformed to HWE.

### Meta-analysis results

**Figure 2:**
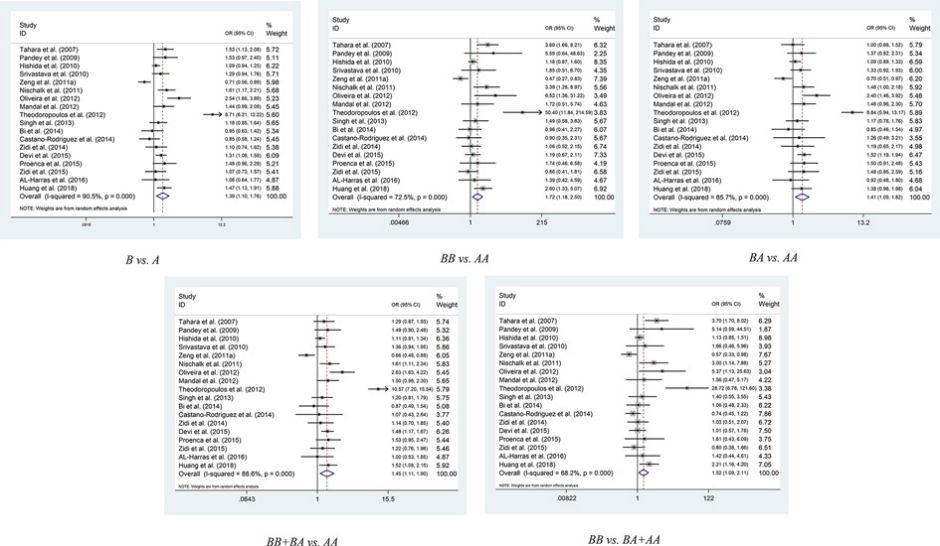
Meta-analysis of the association between *TLR2* -196 to -174 del polymorphism and cancer risk

The results of pooled analysis for *TLR2* polymorphism and cancer susceptibility are provided in Table 2. For -196 to -174del, we collected 18 articles containing 3943 cases and 4574 controls [1–3,6,8–12,28–36]. In the overall analysis, -196 to -174del significantly increased the risk of cancer [B vs. A (OR = 1.468, 95% Cl = 1.129–1.91, *P*=0.005); BB vs. AA (OR = 1.716, 95% Cl = 1.178–2.5, *P*=0.005); BA vs. AA (OR = 1.408, 95% Cl = 1.092–1.816, *P*=0.008); BB+BA vs. AA (OR = 1.449, 95% Cl = 1.107–1.897, *P*=0.007); BB vs. BA+AA (OR = 1.517, 95% Cl = 1.092–2.107, *P*=0.013)] (Figure 2). Among the subgroup of Caucasians, -196 to -174del produces a significant increase in the risk of cancer, too [B vs. A (OR = 3.291, 95% Cl = 1.139–9.51, *P*=0.028); BB vs. AA (OR = 9.878, 95% Cl = 1.83–53.322, *P*=0.008); BA vs. AA (OR = 3.156, 95% Cl = 1.034–9.634, *P*=0.044); BB+BA vs. AA (OR = 3.555, 95% Cl = 1.098–11.51, *P*=0.034); BB vs. BA+AA (OR = 7.294, 95% Cl = 1.752-30.369, *P*=0.006)]. During the subgroup analysis of HB, -196 to -174del was found to be associated with cancer [B vs. A (OR = 1.576, 95% Cl = 1.193–2.08, *P*<0.001); BB vs. AA (OR = 2.274, 95% Cl = 1.43–3.616, *P*<0.001); BA vs. AA (OR = 1.543, 95% Cl = 1.143–2.081, *P*<0.001); BB+BA vs. AA (OR = 1.624, 95% Cl = 1.186–2.223, *P*<0.001); BB vs. BA+AA (OR = 2.011, 95% Cl = 1.317–3.07, *P*=0.001)]. In addition, in the subgroup analysis of Asians, the models of BB+BA vs. AA (OR = 1.203, 95% Cl = 1.015–1.427, *P*=0.033) and B vs. A (OR = 1.169, 95% Cl = 1.005–1.361, *P*=0.043) suggested that -196 to -174del increased the risk of cancer. Meanwhile, when -196 to -174del conformed to HWE in the control group, analysis of all models showed that the deletion of these 22 genes increased the risk of cancer (Supplementary Table S2). By the way, the BA vs. AA model in the N subgroup suggested that -196 to-174del was related to the cancer risk (OR = 1.335, 95% Cl = 1.015–1.757, *P*=0.039).

**Table 2:** Results of pooled analysis for *TLR2* polymorphism and cancer susceptibility

Comparison	Subgroup	*n*	Cases	Controls	*P*_H_	*P*_Z_	HR (95% CI)
(-196 to -174del)							
B vs. A	Overall	18	3943	6394	<0.001	0.005*	1.468 (1.129–1.91)
BB vs. AA	Overall	18	3943	6394	<0.001	0.005*	1.716 (1.178–2.5)
BA vs. AA	Overall	18	3943	6394	<0.001	0.008*	1.408 (1.092–1.816)
BB+BA vs. AA	Overall	18	3943	6394	<0.001	0.007*	1.449 (1.107–1.897)
BB vs. BA+ AA	Overall	18	3943	6394	<0.001	0.013*	1.517 (1.092–2.107)
B vs. A	Asian	11	2807	4482	<0.001	0.043*	1.169 (1.005–1.361)
BB vs. AA	Asian	11	2807	4482	0.003	0.098	1.373 (0.943–2)
BA vs. AA	Asian	11	2807	4482	0.039	0.054	1.168 (0.997–1.367)
BB+BA vs. AA	Asian	11	2807	4482	0.008	0.033*	1.203 (1.015–1.427)
BB vs. BA+ AA	Asian	11	2807	4482	0.005	0.177	1.256 (0.902–1.748)
B vs. A	Caucasian	3	624	1052	<0.001	0.028*	3.291 (1.139–9.51)
BB vs. AA	Caucasian	3	624	1052	0.007	0.008*	9.878 (1.83–53.322)
BA vs. AA	Caucasian	3	624	1052	<0.001	0.044*	3.156 (1.034–9.634)
BB+BA vs. AA	Caucasian	3	624	1052	<0.001	0.034*	3.555 (1.098–11.51)
BB vs. BA+ AA	Caucasian	3	624	1052	0.029	0.006*	7.294 (1.752–30.369)
B vs. A	African	4	512	860	0.653	0.159	1.163 (0.943–1.436)
BB vs. AA	African	4	512	860	0.796	0.746	1.076 (0.693–1.67)
BA vs. AA	African	4	512	860	0.652	0.075	1.296 (0.974–1.724)
BB+BA vs. AA	African	4	512	860	0.72	0.106	1.232 (0.956–1.586)
BB vs. BA+AA	African	4	512	860	0.755	0.897	1.029 (0.666–1.59)
B vs. A	PB	14	2904	3782	<0.001	0.001*	1.576 (1.193–2.08)
BB vs. AA	PB	14	2904	3782	<0.001	0.001*	2.274 (1.43–3.616)
BA vs. AA	PB	14	2904	3782	<0.001	0.005*	1.543 (1.143–2.081)
BB+BA vs. AA	PB	14	2904	3782	<0.001	0.002*	1.624 (1.186–2.223)
BB vs. BA+AA	PB	14	2904	3782	0.001	0.001*	2.011 (1.317–3.07)
B vs. A	HB	4	1039	2612	0.016	0.502	0.92 (0.721–1.173)
BB vs. AA	HB	4	1039	2612	0.048	0.552	0.866 (0.54–1.39)
BA vs. AA	HB	4	1039	2612	0.122	0.841	0.984 (0.837–1.156)
BB+BA vs. AA	HB	4	1039	2612	0.038	0.716	0.942 (0.684–1.298)
BB vs. BA+AA	HB	4	1039	2612	0.121	0.43	0.917 (0.739–1.138)
B vs. A	Gastric cancer	6	1640	2983	<0.001	0.194	1.22 (0.904–1.647)
BB vs. AA	Gastric cancer	6	1640	2983	<0.001	0.176	1.565 (0.818–2.995)
BA vs. AA	Gastric cancer	6	1640	2983	0.002	0.309	1.171 (0.864–1.586)
BB+BA vs. AA	Gastric cancer	6	1640	2983	<0.001	0.216	1.246 (0.879–1.764)
BB vs. BA+AA	Gastric cancer	6	1640	2983	<0.001	0.223	1.401 (0.814–2.411)
B vs. A	Breast cancer	3	795	1350	<0.001	0.212	2.31 (0.621–8.593)
BB vs. AA	Breast cancer	3	795	1350	<0.001	0.2	4.049 (0.478–34.306)
BA vs. AA	Breast cancer	3	795	1350	<0.001	0.197	2.347 (0.642–8.58)
BB+BA vs. AA	Breast cancer	3	795	1350	<0.001	0.2	2.52 (0.613–10.36)
BB vs. BA+AA	Breast cancer	3	795	1350	<0.001	0.233	3.176 (0.476–21.196)
B vs. A	Cervical cancer	4	504	770	0.474	0.269	1.121 (0.916–1.372)
BB vs. AA	Cervical cancer	4	504	770	0.453	0.782	1.061 (0.696–1.618)
BA vs. AA	Cervical cancer	4	504	770	0.554	0.177	1.215 (0.916–1.613)
BB+BA vs. AA	Cervical cancer	4	504	770	0.586	0.207	1.177 (0.914–1.515)
BB vs. BA+AA	Cervical cancer	4	504	770	0.456	0.848	1.041 (0.692–1.566)
B vs. A	Y	15	3459	5620	<0.001	0.008*	1.447 (1.103–1.897)
BB vs. AA	Y	15	3459	5620	<0.001	0.004*	1.915 (1.227–2.991)
BA vs. AA	Y	15	3459	5620	<0.001	0.02*	1.422 (1.057–1.915)
BB+BA vs. AA	Y	15	3459	5620	<0.001	0.013*	1.494 (1.088–2.052)
BB vs. BA+AA	Y	15	3459	5620	<0.001	0.009*	1.673 (1.137–2.461)
B vs. A	N	3	484	774	0.709	0.14	1.168 (0.951–1.434)
BB vs. AA	N	3	484	774	0.597	0.84	1.05 (0.655–1.681)
BA vs. AA	N	3	484	774	0.872	0.039*	1.335 (1.015–1.757)
BB+BA vs. AA	N	3	484	774	0.839	0.07	1.258 (0.981–1.613)
BB vs. BA+AA	N	3	484	774	0.615	0.959	0.988 (0.62–1.575)
rs3804099							
B vs. A	Overall	9	1901	2618	0.001	0.723	0.967 (0.806–1.162)
BB vs. AA	Overall	9	1901	2618	0.029	0.29	0.84 (0.609–1.16)
BA vs. AA	Overall	9	1901	2618	0.643	0.008*	0.827 (0.717–0.952)
BB+BA vs. AA	Overall	9	1901	2618	0.446	0.016*	0.85 (0.744–0.97)
BB vs. BA+AA	Overall	10	3456	4574	0.001	0.946	0.991 (0.768–1.28)
B vs. A	Asian	5	783	1288	0.013	0.177	0.838 (0.648–1.083)
BB vs. AA	Asian	5	783	1288	0.721	0.005*	0.65 (0.482–0.877)
BA vs. AA	Asian	5	783	1288	0.892	0.001*	0.69 (0.55–0.867)
BB+BA vs. AA	Asian	5	783	1288	0.994	<0.001	0.684 (0.555–0.843)
BB vs. BA+AA	Asian	5	783	1288	0.005	0.559	0.869 (0.542–1.393)
B vs. A	Caucasian	4	1118	1330	0.025	0.3	1.147 (0.885–1.486)
BB vs. AA	Caucasian	4	1118	1330	0.024	0.455	1.283 (0.667–2.47)
BA vs. AA	Caucasian	4	1118	1330	0.819	0.425	0.929 (0.774–1.114)
BB+BA vs. AA	Caucasian	4	1118	1330	0.87	0.866	0.985 (0.829–1.171)
BB vs. BA+AA	Caucasian	5	2673	3286	0.01	0.647	1.082 (0.771–1.518)
B vs. A	Breast cancer	2	214	235	0.647	0.364	0.885 (0.68–1.152)
BB vs. AA	Breast cancer	2	214	235	0.611	0.399	0.796 (0.47–1.351)
BA vs. AA	Breast cancer	2	214	235	0.887	0.302	0.792 (0.509–1.233)
BB+BA vs. AA	Breast cancer	2	214	235	0.765	0.276	0.793 (0.523–1.203)
BB vs. BA+AA	Breast cancer	2	214	235	0.621	0.713	0.921 (0.592–1.432)
B vs. A	Gastric Cancer	2	336	808	0.831	0.002*	0.728 (0.594–0.893)
BB vs. AA	Gastric Cancer	2	336	808	0.75	0.026*	0.605 (0.389–0.942)
BA vs. AA	Gastric Cancer	2	336	808	0.926	0.018*	0.706 (0.529–0.942)
BB+BA vs. AA	Gastric Cancer	2	336	808	0.956	0.004*	0.681 (0.524–0.886)
BB vs. BA+AA	Gastric Cancer	2	336	808	0.928	0.083	0.683 (0.444–1.051)
BB vs. BA+ AA	Colon Cancer	2	1666	2058	0.243	0.034*	0.841 (0.716–0.987)
B vs. A	PB	5	1172	1400	0.004	0.985	0.997 (0.759–1.311)
BB vs. AA	PB	5	1172	1400	0.01	0.762	0.912 (0.502–1.658)
BA vs. AA	PB	5	1172	1400	0.764	0.252	0.901 (0.754–1.077)
BB+BA vs. AA	PB	5	1172	1400	0.468	0.385	0.928 (0.785–1.098)
BB vs. BA+AA	PB	6	2727	3356	0.021	0.549	0.915 (0.683–1.225)
B vs. A	HB	4	729	1218	0.007	0.658	0.934 (0.691–1.263)
BB vs. AA	HB	4	729	1218	0.29	0.155	0.794 (0.577–1.091)
BA vs. AA	HB	4	729	1218	0.624	0.005*	0.713 (0.564–0.902)
BB+BA vs. AA	HB	4	729	1218	0.679	0.005*	0.734 (0.591–0.912)
BB vs. BA+AA	HB	4	729	1218	0.012	0.782	1.073 (0.65–1.772)
B vs. A	Y	5	1327	1792	0.13	0.036*	0.895 (0.807–0.993)
BB vs. AA	Y	5	1327	1792	0.233	0.087	0.828 (0.668–1.028)
BA vs. AA	Y	5	1327	1792	0.484	0.058	0.856 (0.729–1.005)
BB+BA vs. AA	Y	5	1327	1792	0.258	0.028*	0.844 (0.725–0.982)
BB vs. BA+ AA	Y	5	1327	1792	0.437	0.265	0.898 (0.742–1.086)
B vs. A	N	4	574	826	0.004	0.37	1.179 (0.823–1.688)
BB vs. AA	N	4	574	826	0.008	0.596	1.262 (0.534–2.98)
BA vs. AA	N	4	574	826	0.628	0.042*	0.73 (0.54–0.988)
BB+BA vs. AA	N	4	574	826	0.469	0.315	0.87 (0.663–1.142)
BB vs. BA+AA	N	4	574	826	0.002	0.242	1.564 (0.739–3.308)
rs3804100							
B vs. A	Overall	8	2842	3081	0.422	0.254	1.076 (0.949–1.219)
BB vs. AA	Overall	8	2842	3081	0.682	0.412	0.823 (0.516–1.311)
BA vs. AA	Overall	8	2842	3081	0.487	0.603	1.041 (0.896–1.209)
BB+BA vs. AA	Overall	8	2842	3081	0.758	0.641	1.035 (0.894–1.199)
BB vs. BA+AA	Overall	8	2842	3081	0.243	0.061	1.343 (0.987–1.827)
B vs. A	Asian	5	622	1005	0.152	0.71	1.037 (0.856–1.257)
BB vs. AA	Asian	5	622	1005	0.66	0.153	0.655 (0.366–1.17)
BA vs. AA	Asian	5	622	1005	0.276	0.543	0.917 (0.692–1.213)
BB+BA vs. AA	Asian	5	622	1005	0.688	0.391	0.888 (0.677–1.165)
BB vs. BA+AA	Asian	5	622	1005	0.105	0.079	1.346 (0.966–1.875)
B vs. A	Caucasian	3	2220	2076	0.937	0.237	1.105 (0.937–1.304)
BB vs. AA	Caucasian	3	2220	2076	0.618	0.494	1.337 (0.582–3.075)
BA vs. AA	Caucasian	3	2220	2076	0.87	0.317	1.095 (0.917–1.308)
BB+BA vs. AA	Caucasian	3	2220	2076	0.908	0.268	1.104 (0.927–1.315)
BB vs. BA+AA	Caucasian	3	2220	2076	0.612	0.51	1.323 (0.576–3.039)
B vs. A	PB	4	2269	2142	0.365	0.555	1.049 (0.896–1.228)
BB vs. AA	PB	4	2269	2142	0.471	0.91	0.959 (0.465–1.977)
BA vs. AA	PB	4	2269	2142	0.402	0.495	1.061 (0.894–1.26)
BB+BA vs. AA	PB	4	2269	2142	0.384	0.514	1.057 (0.894–1.251)
BB vs. BA+ AA	PB	4	2269	2142	0.479	0.911	0.96 (0.466–1.978)
B vs. A	HB	4	573	939	0.308	0.266	1.124 (0.915–1.381)
BB vs. AA	HB	4	573	939	0.512	0.336	0.74 (0.4–1.368)
BA vs. AA	HB	4	573	939	0.346	0.872	0.975 (0.715–1.329)
BB+BA vs. AA	HB	4	573	939	0.83	0.829	0.967 (0.715–1.308)
BB vs. BA+AA	HB	4	573	939	0.146	0.033*	1.449 (1.031–2.036)
B vs. A	Breast cancer	2	213	241	0.429	0.886	0.968 (0.617–1.517)
BA vs. AA	Breast cancer	2	213	241	0.663	0.662	1.118 (0.679–1.839)
BB+BA vs. AA	Breast cancer	2	213	241	0.533	0.867	1.042 (0.641–1.695)
B vs. A	Gastric cancer	2	173	524	0.493	0.598	0.918 (0.669–1.261)
BB vs. AA	Gastric cancer	2	173	524	0.259	0.168	0.481 (0.17–1.362)
BA vs. AA	Gastric cancer	2	173	524	0.88	0.531	1.129 (0.772–1.652)
BB+BA vs. AA	Gastric cancer	2	173	524	0.675	0.927	1.018 (0.703–1.473)
BB vs. BA+AA	Gastric cancer	2	173	524	0.27	0.142	0.463 (0.165–1.295)
B vs. A	Y	6	2543	2537	0.666	0.546	1.045 (0.905–1.207)
BB vs. AA	Y	6	2543	2537	0.706	0.824	0.935 (0.516–1.695)
BA vs. AA	Y	6	2543	2537	0.683	0.436	1.065 (0.909–1.248)
BB+BA vs. AA	Y	6	2543	2537	0.688	0.467	1.059 (0.907–1.237)
BB vs. BA+AA	Y	6	2543	2537	0.693	0.771	0.916 (0.508–1.653)
B vs. A	N	2	299	544	0.075	0.741	1.091 (0.652–1.824)
BB vs. AA	N	2	299	544	0.188	0.308	0.674 (0.316–1.439)
BA vs. AA	N	2	299	544	0.108	0.507	0.855 (0.537–1.36)
BB+BA vs. AA	N	2	299	544	0.563	0.499	0.855 (0.543–1.346)
BB vs. BA+AA	N	2	299	544	0.073	0.789	0.716 (0.062–8.24)
rs4696480							
B vs. A	Overall	4	492	500	0.323	0.03*	1.216 (1.019–1.452)
BB vs. AA	Overall	4	492	500	0.344	0.032*	1.463 (1.034–2.069)
BA vs. AA	Overall	4	492	500	0.059	0.167	1.407 (0.867–2.281)
BB+BA vs. AA	Overall	4	492	500	0.076	0.115	1.415 (0.919–2.179)
BB vs. BA+AA	Overall	4	492	500	0.836	0.296	1.169 (0.872–1.568)
B vs. A	Asian	2	232	230	0.628	0.772	1.039 (0.801–1.348)
BB vs. AA	Asian	2	232	230	0.563	0.692	1.106 (0.671–1.824)
BA vs. AA	Asian	2	232	230	0.711	0.77	0.939 (0.616–1.433)
BB+BA vs. AA	Asian	2	232	230	0.981	0.968	0.992 (0.672–1.465)
BB vs. BA+AA	Asian	2	232	230	0.382	0.596	1.125 (0.728–1.738)
B vs. A	Caucasian	2	260	270	0.424	0.007*	1.393 (1.094–1.775)
BB vs. AA	Caucasian	2	260	270	0.406	0.009*	1.903 (1.171–3.091)
BA vs. AA	Caucasian	2	260	270	0.252	0.001*	1.984 (1.307–3.012)
BB+BA vs. AA	Caucasian	2	260	270	0.261	0.001*	1.95 (1.317–2.887)
BB vs. BA+AA	Caucasian	2			0.848	0.351	1.208 (0.812–1.798)
B vs. A	PB	3	307	319	0.21	0.176	1.167 (0.933–1.458)
BB vs. AA	PB	3	307	319	0.217	0.152	1.369 (0.891–2.105)
BA vs. AA	PB	3	307	319	0.044	0.421	1.322 (0.67–2.611)
BB+BA vs. AA	PB	3	307	319	0.056	0.349	1.336 (0.729–2.449)
BB vs. BA+AA	PB	3	307	319	0.652	0.408	1.167 (0.809–1.681)
B vs. A	Y	3	417	411	0.463	0.158	1.15 (0.947–1.396)
BB vs. AA	Y	3	417	411	0.502	0.163	1.31 (0.897–1.916)
BA vs. AA	Y	3	417	411	0.183	0.238	1.211 (0.881–1.665)
BB+BA vs. AA	Y	3	417	411	0.227	0.158	1.239 (0.921–1.666)
BB vs. BA+AA	Y	3	427	411	0.677	0.412	1.146 (0.827–1.588)
rs5743708							
B vs. A	Overall	2	309	572	<0.001	0.321	4.076 (0.255–65.24)
BA vs. AA	Overall	2	309	572	0.022	0.338	1.697 (0.575–5.011)
BB+BA vs. AA	Overall	4	2618	3487	<0.001	0.312	1.651 (1.348–2.022)
rs1898830							
B vs. A	Overall	3	2520	3146	0.391	0.939	1.003 (0.928–1.085)
BB vs. AA	Overall	3	2520	3146	0.323	0.646	0.961 (0.809–1.14)
BA vs. AA	Overall	3	2520	3146	0.056	0.806	0.971 (0.768–1.227)
BB+BA vs. AA	Overall	3	2520	3146	0.075	0.813	0.975 (0.791–1.202)
BB vs. BA+AA	Overall	3	2520	3146	0.998	0.77	0.977 (0.835–1.143)
B vs. A	Caucasian	2	2309	2914	0.623	0.655	1.019 (0.939–1.106)
BB vs. AA	Caucasian	2	2309	2914	0.779	0.972	1.003 (0.837–1.202)
BA vs. AA	Caucasian	2	2309	2914	0.515	0.355	1.056 (0.941–1.187)
BB+BA vs. AA	Caucasian	2	2309	2914	0.518	0.433	1.045 (0.936–1.167)
BB vs. BA+AA	Caucasian	2	2309	2914	0.955	0.777	0.975 (0.822–1.158)
B vs. A	PB	2	2309	2914	0.623	0.655	1.019 (0.939–1.106)
BB vs. AA	PB	2	2309	2914	0.779	0.972	1.003 (0.837–1.202)
BA vs. AA	PB	2	2309	2914	0.515	0.355	1.056 (0.941–1.187)
BB+BA vs. AA	PB	2	2309	2914	0.518	0.433	1.045 (0.936–1.167)
BB vs. BA+AA	PB	2	2309	2914	0.955	0.777	0.975 (0.822–1.158)

Abbreviations: *n*, polymorphisms did not conform to HWE in the control group; P-B, population based; *P_H_*, *P*-value of Q test for heterogeneity test; *P*_Z_, means statistically significant (*P*<0.05); Y, polymorphisms conformed to HWE in the control group. * *P*-value less than 0.05 was considered as statistically significant.

**Figure 3:**
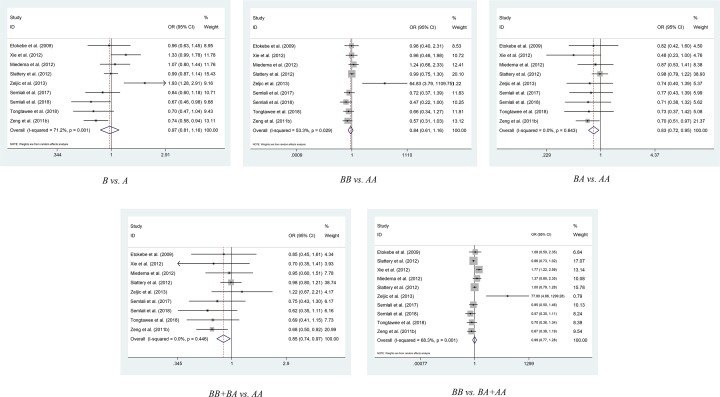
Meta-analysis of the association between *TLR2* rs3804009 del polymorphism and cancer risk

There are nine studies on rs3804099 polymorphism including a total of 3456 cases and 4574 controls [13–16,18,37–40]. According to overall analysis, rs3804099 significantly decreased cancer risk [BA vs. AA (OR = 0.827, 95% Cl = 0.717–0.952, *P*=0.008), BB+BA vs. AA (OR = 0.85, 95% Cl = 0.744–0.97, *P*=0.016)] (Figure 3). About Asians, rs3804099 polymorphism reduced the risk of cancer in the model of BA vs. AA (OR = 0.69, 95% Cl = 0.55–0.867, *P*=0.001) and BB vs. AA (OR = 0.65, 95% Cl = 0.482–0.877, *P*=0.005). In the subgroup of gastric cancer patients, we found that rs3804099 polymorphism reduced the risk of cancer [B vs. A (OR = 0.728, 95% Cl = 0.594–0.893, *P*=0.002), BB vs. AA (OR = 0.605, 95% Cl = 0.389–0.942, *P*=0.026), BA vs. AA (OR = 0.706, 95% Cl = 0.529–0.942, *P*=0.018), BB+BA vs. AA (OR = 0.681, 95% Cl = 0.524–0.886, *P*=0.004)] and the model of BB vs. BA+AA is not associated with reduced risk of gastric cancer. Part of the model in the hospital-based analysis was associated with reduced cancer risk [BA vs. AA (OR = 0.713, 95% Cl = 0.564–0.902, *P*=0.005), BB+BA vs. AA (OR = 0.734, 95% Cl = 0.591–0.912, *P*=0.005)].

There are four studies on rs4696480 polymorphism including a total of 492 cases and 500 controls [14,17,18,38]. In some models of the overall analysis, rs4696480 significantly increased cancer risk [B vs. A (OR = 1.216, 95% Cl = 1.019–1.452, *P*=0.03); BB vs. AA (OR = 1.463, 95% Cl = 1.034–2.069, *P*=0.032)]. It is worth mentioning that rs4696480 makes Caucasians more susceptible to cancer [B vs. A (OR = 1.393, 95% Cl = 1.094–1.775, *P*=0.007), BB vs. AA (OR = 1.903, 95% Cl = 1.171–3.091, *P*=0.009), BA vs. AA (OR = 1.984, 95% Cl = 1.307–3.012, *P*=0.001), BB+BA vs. AA (OR = 1.95, 95% Cl = 1.317–2.887, *P*=0.001)]. Thus, we can conclude that a subgroup analysis by ethnicity suggests that rs4696480 is related to cancer risk in Caucasians, but not in other ethnic groups (Table 2 and Supplementary Figure S1).

For rs3804100 polymorphism, we collected eight publications which contained 2842 cases and 3081 controls [1,13–16,18,38,41]. But only in hospital-based analysis we found the model of BB vs. BA+AA (OR = 1.449, 95% Cl = 1.031–2.036, *P*=0.033) added to the risk of cancer. None of the other models showed any association between rs3804100 and cancer risk, either in the analysis of overall group or in other subgroups (Table 2 and Supplementary Figure S2).

As for rs5743708 [6,37,42] and rs1898830 [16,37], they were found to have no significant correlation with cancer, either in overall analysis or in other subgroup analysis (Table 2 and Supplementary Figures S3 and S4).

### Sensitivity analysis and publication bias

By the way, we removed individual study one by one when conducted the sensitivity analysis. We did not observe any significant changes in the OR and corresponding 95% CI values, so the stability of our results was confirmed. All the details of sensitivity analysis are shown in the Supplementary Table S2 and Figure S5.

**Figure 4:**
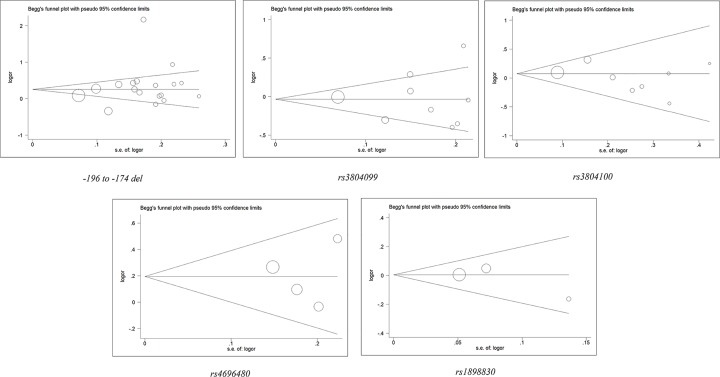
Begg’s funnel plot for *TLR2* polymorphisms and overall cancer publication bias (B vs. A) For Begg’s funnel plot, the x-axis is log (OR), and the y-axis is natural logarithm of OR. The horizontal line in the figure represents the overall estimated log (OR). The two diagonal lines indicate the pseudo 95% confidence limits of the effect estimate.

We used the Begg’s test to evaluate publication bias for selected literature. These funnel plots in Figure 4 showed the relationship between the cancer risk and the *TLR2* polymorphism in this meta-analysis. Among the various polymorphic sites, the funnel plots were symmetrically distributed. This showed that there was no publication bias. The Egger’s test further analyzed the publication bias, and showed that no significant evidence of publication bias was observed in our study (*P*=0.937 for SNP rs4696480; *P*=0.291 for - 196 to - 174del polymorphism; *P*=0.991 for SNP rs3804099) (Supplementary Table S3).

## Results of FPRP and TSA

**Figure 5:**
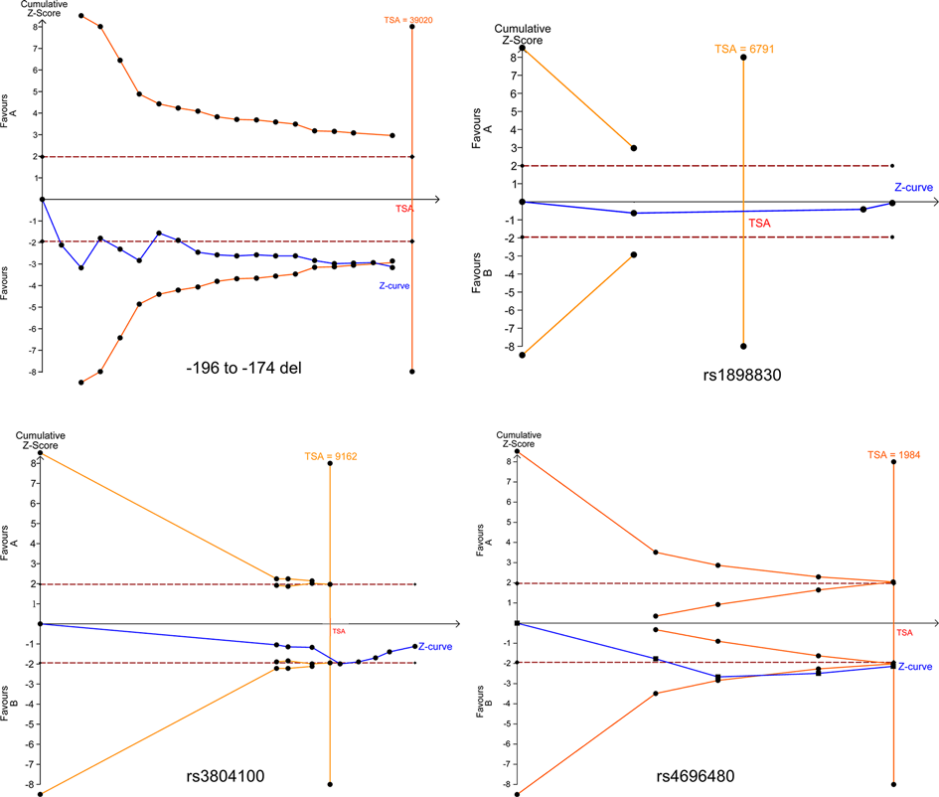
TSA for *TLR2* polymorphism under the allele contrast model (B vs. A)

The FPRP values for positive findings at different prior probability levels are shown in Table 3. For -196 to -174del variant, almost all the statistical power high than 0.2, for the FPRP values, under the prior probability of 0.25, the FPRP values for each group is less than 0.2, except the five genetic models about Caucasian subgroup. Which means that the results on Caucasian subgroup are not stable, more studies are needed to illustrate the results. For the other positive results on rs3804099, rs3804100 and rs4696480, almost all the statistical power was higher than 0.5, and under the prior probability of 0.25, the FPRP values for each group is less than 0.2, which means that the results are reliable. The results of TSA are shown in Figure 5, we analyzed the required sample size of each polymorphism. The required sample size of -196 to -174del variant is approximately 39020, although the sample size in the current study did not meet the required number, we observed that the cumulative z-curve crossed the trial sequential monitoring boundary and the traditional significant boundary (Z = 1.96, α = 0.05), which means that our conclusions were robust with the sufficient evidence. For rs3804100 (required sample size: 9162) and rs4696480 (required sample size: 1984), we observed that the cumulative z-curve crossed the trial sequential monitoring boundary and the traditional significant boundary, and meet the required number. The TSA result about rs1898830 showed that the mutant allele performed the similar impact on cancer risk compare with the wild allele, no more samples are needed to confirm the result (Figure 5). However, The TSA results of rs3804099 and rs5743708 indicated that more objects are need to drag out the robust conclusion (Supplementary Figure S6).

**Table 3:** FPRP values for associations between the risk of cancer and the frequency of genotypes

Comparison	Subgroup	*n*	P_Z_	OR (95% CI)	Statistical power				
						0.25	0.1	0.01	0.001
**(-196 to -174del)**									
B vs. A	Overall	18	0.005*	1.468 (1.129–1.91)	0.564	0.022^†^	0.064^†^	0.427	0.883
BB vs. AA	Overall	18	0.005*	1.716 (1.178–2.5)	0.237	0.054^†^	0.146^†^	0.652	0.950
BA vs. AA	Overall	18	0.008*	1.408 (1.092–1.816)	0.683	0.035^†^	0.099^†^	0.547	0.924
BB+BA vs. AA	Overall	18	0.007*	1.449 (1.107–1.897)	0.597	0.034^†^	0.096^†^	0.539	0.922
BB vs. BA+ AA	Overall	18	0.013*	1.517 (1.092–2.107)	0.468	0.073^†^	0.192^†^	0.723	0.963
B vs. A	Asian	11	0.043*	1.169 (1.005–1.361)	0.999	0.117^†^	0.285	0.814	0.978
BB+BA vs. AA	Asian	11	0.033*	1.203 (1.015–1.427)	0.994	0.106^†^	0.262	0.796	0.975
B vs. A	Caucasian	3	0.028*	3.291 (1.139–9.51)	0.073	0.532	0.773	0.974	0.997
BB vs. AA	Caucasian	3	0.008*	9.878 (1.83–53.322)	0.014	0.621	0.831	0.982	0.998
BA vs. AA	Caucasian	3	0.044*	3.156 (1.034–9.634)	0.096	0.577	0.804	0.978	0.998
BB+BA vs. AA	Caucasian	3	0.034*	3.555 (1.098–11.51)	0.075	0.579	0.805	0.978	0.998
BB vs. BA+ AA	Caucasian	3	0.006*	7.294 (1.752–30.369)	0.015	0.561	0.793	0.977	0.998
B vs. A	PB	14	0.001*	1.576 (1.193–2.08)	0.364	0.011^†^	0.031^†^	0.263	0.783
BB vs. AA	PB	14	0.001*	2.274 (1.43–3.616)	0.040	0.039^†^	0.108^†^	0.571	0.931
BA vs. AA	PB	14	0.005*	1.543 (1.143–2.081)	0.427	0.031^†^	0.086^†^	0.510	0.913
BB+BA vs. AA	PB	14	0.002*	1.624 (1.186–2.223)	0.310	0.023^†^	0.067^†^	0.441	0.888
BB vs. BA+ AA	PB	14	0.001*	2.011 (1.317–3.07)	0.087	0.040^†^	0.111^†^	0.578	0.933
B vs. A	Y	15	0.008*	1.447 (1.103–1.897)	0.603	0.036^†^	0.101^†^	0.551	0.925
BB vs. AA	Y	15	0.004*	1.915 (1.227–2.991)	0.141	0.083^†^	0.214	0.750	0.968
BA vs. AA	Y	15	0.02*	1.422 (1.057–1.915)	0.637	0.088^†^	0.224	0.760	0.970
BB+BA vs. AA	Y	15	0.013*	1.494 (1.088–2.052)	0.510	0.072^†^	0.189	0.719	0.963
BB vs. BA+ AA	Y	15	0.009*	1.673 (1.137–2.461)	0.290	0.085^†^	0.218	0.754	0.969
BA vs. AA	N	3	0.039*	1.335 (1.015–1.757)	0.797	0.129^†^	0.307	0.830	0.980
**rs3804099**									
BA vs. AA	Overall	9	0.008*	0.827 (0.717–0.952)	0.999	0.024^†^	0.069^†^	0.448	0.891
BB+BA vs. AA	Overall	9	0.016*	0.85 (0.744–0.97)	1.000	0.045^†^	0.125^†^	0.611	0.941
BB vs. AA	Asian	5	0.005*	0.65 (0.482–0.877)	0.434	0.032^†^	0.091^†^	0.524	0.917
BA vs. AA	Asian	5	0.001*	0.69 (0.55–0.867)	0.287	0.064^†^	0.170^†^	0.692	0.958
B vs. A	Gastric cancer	2	0.002*	0.728 (0.594–0.893)	0.801	0.009^†^	0.025^†^	0.223	0.743
BB vs. AA	Gastric cancer	2	0.026*	0.605 (0.389–0.942)	0.334	0.190^†^	0.413	0.886	0.987
BA vs. AA	Gastric cancer	2	0.018*	0.706 (0.529–0.942)	0.652	0.076^†^	0.199^†^	0.732	0.965
BB+BA vs. AA	Gastric cancer	2	0.004*	0.681 (0.524–0.886)	0.563	0.022^†^	0.063^†^	0.426	0.882
BB vs. BA+ AA	Colon cancer	2	0.034*	0.841 (0.716-0.987)	0.998	0.093^†^	0.235	0.771	0.971
BA vs. AA	HB	4	0.005*	0.713 (0.564–0.902)	0.712	0.020^†^	0.057^†^	0.400	0.871
BB+BA vs. AA	HB	4	0.005*	0.734 (0.591–0.912)	0.807	0.019^†^	0.055^†^	0.391	0.867
B vs. A	Y	5	0.036*	0.895 (0.807–0.993)	1.000	0.098^†^	0.247	0.783	0.973
BB+BA vs. AA	Y	5	0.028*	0.844 (0.725–0.982)	0.999	0.078^†^	0.202	0.736	0.966
BA vs. AA	N	4	0.042*	0.73 (0.54–0.988)	0.722	0.147^†^	0.341	0.851	0.983
**rs3804100**									
BB vs. BA+ AA	HB	4	0.033*	1.449 (1.031–2.036)	0.579	0.144^†^	0.336	0.848	0.983
**rs4696480**									
B vs. A	Overall	4	0.03*	1.216 (1.019–1.452)	0.990	0.085^†^	0.218	0.754	0.969
BB vs. AA	Overall	4	0.032*	1.463 (1.034–2.069)	0.556	0.145^†^	0.337	0.848	0.983
B vs. A	Caucasian	2	0.007*	1.393 (1.094–1.775)	0.725	0.029^†^	0.084^†^	0.501	0.910
BB vs. AA	Caucasian	2	0.009*	1.903 (1.171–3.091)	0.168	0.143^†^	0.333	0.846	0.982
BA vs. AA	Caucasian	2	0.001*	1.984 (1.307–3.012)	0.095	0.040^†^	0.110^†^	0.576	0.932
BB+BA vs. AA	Caucasian	2	0.001*	1.95 (1.317–2.887)	0.095	0.026^†^	0.075^†^	0.470	0.899

Statistical power was calculated using the number of observations in the subgroup and the OR and *P* values in this table. Abbreviations: CI, confidence interval; H-B, hospital based; HWE (Y), polymorphisms conformed to HWE in the control group. *P-value less than 0.05 was considered as statistically significant. ^†^The significant result with the FPRP values less than 0.2 was considered a worthy finding.

### LD analyses and *in-silico* analysis of *TLR2* expression

**Figure 6:**
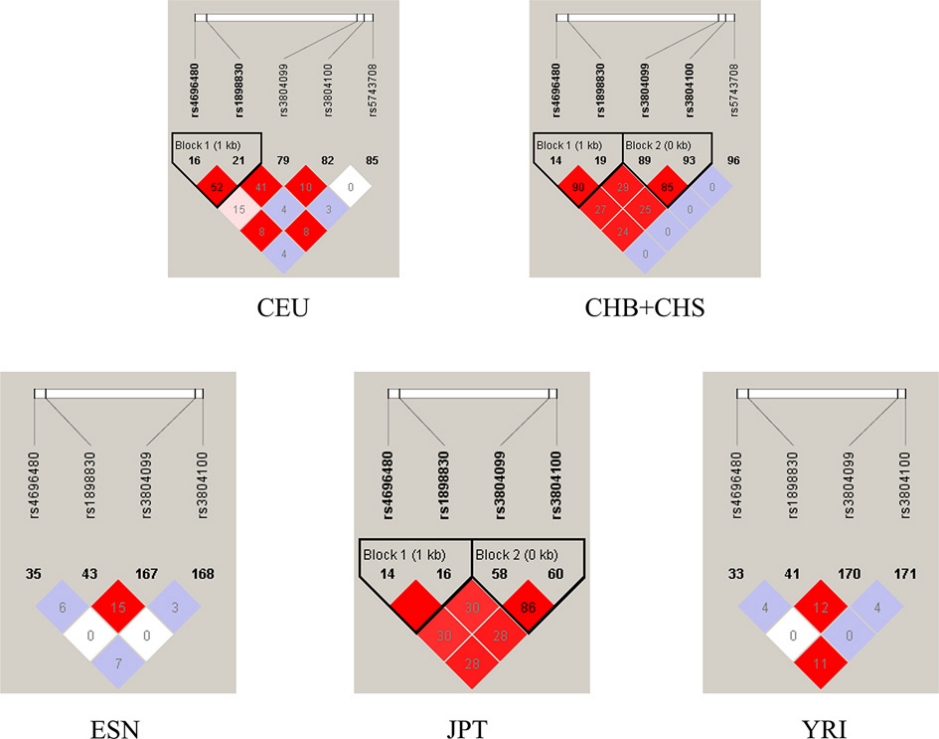
LD analyses for TLR2 polymorphisms in populations from 1000 genomes Phase 3 The number of each cell represents r^2^ and white color cells show no LD between polymorphisms.

**Figure 7:**
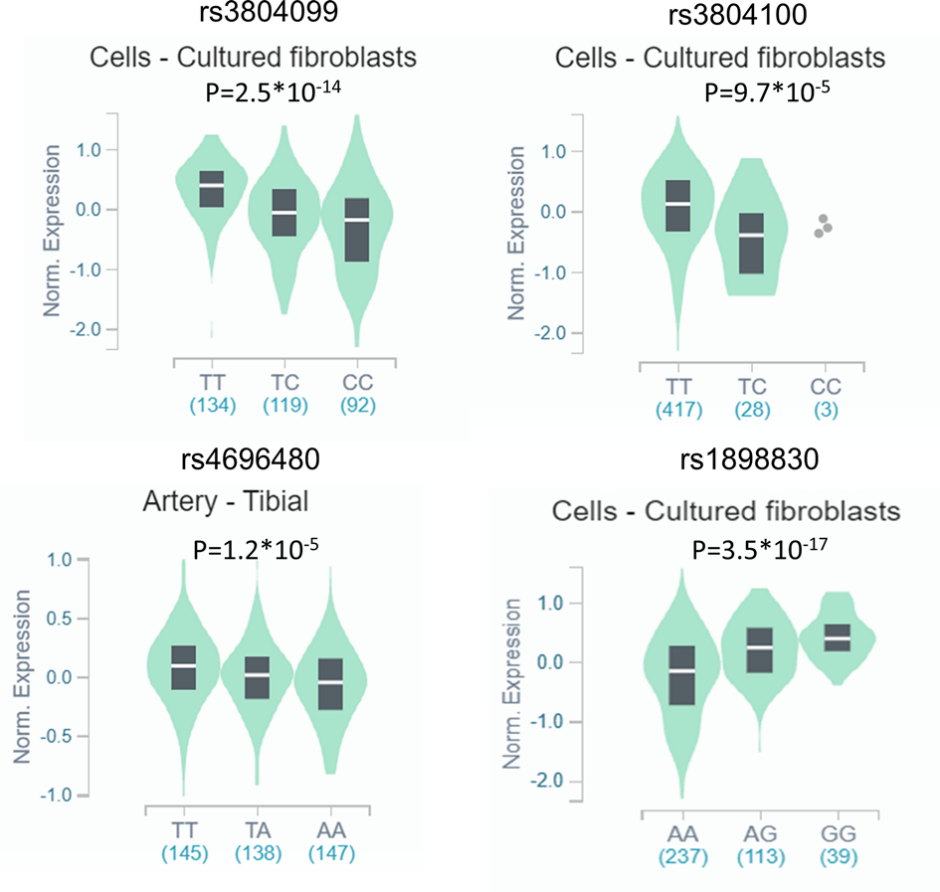
*In-silico* analysis of TLR2 expression concerned to its polymorphisms

LD analysis was conducted to evaluate the presence of bins in different *TLR2* polymorphisms, aiming to understand the internal linkages, the results of which are shown in Figure 6. Highlighted, there is significant LD between rs4696480 and rs1898830 in CEU, CHB and CHS, and JPT populations (CEU: r^2^ = 0.52; CHB and CHS: r^2^ = 0.90; JPT: r^2^ = 1.0). The LD between rs3804099 and rs3804100 is also remarkable in CHB and CHS and JPT populations (CHB and CHS: r^2^ = 0.85; JPT: r^2^ = 0.86) (Supplementary Table S4). According to the result on GTEx portal data, we found that the mutant allele leads to an increase expression of TLR2 mRNA in rs1898830 (*P*=3.5*10^−17^), while the mutant allele of rs3804099 (*P*=2.5*10^−14^), rs3804100 (*P*=9.7*10^−5^) and rs4696480 (*P*=1.2*10^−5^) lead to a decreased expression of TLR2 (Figure 7).

## Discussion

TLRs are expressed in mast cells and several other cell types, which could recognize microbial components and trigger inflammatory response. *TLR2* is type I transmembrane transporter which plays an important role in immune inflammatory response [43], and have been shown to influence host defense and disease progression [44]. There have been four previous meta-analyses on *TLR2*. But two of the studies were limited to gastric cancer [45,46]. One of these articles suggested that - 196 to - 174del was associated with the rise of cancer risk and the rs3804099 can decrease cancer risk [47]. Another article suggested that -196 to -174del had no relationship with cervical cancer [48]. For assessing the real influence of *TLR2* on cancer risk, we collected more samples than before. And our meta-analysis combines many types of cancers to study the relationship between *TLR2* polymorphism and cancer risk as comprehensively as possible.

For -196 to -174del, it is a 22-bp deletion at the promoter region of *TLR2* gene. Transcriptional reduction in the *TLR2* gene due to this substitution may significantly alter the function of the promoter [49]. Chen et al.’s meta-analysis [45] thought that this polymorphism is not associated with gastric cancer. Yang et al. [48] published a meta-analysis in 2018 suggesting that -196 to -174del had nothing to do with cervical cancer. And in our calculations, we revealed that the deletion of these 22 genes does increase the risk of cancer, especially among Caucasians. However, the subgroup calculations of gastric, breast and cervical cancers had no obvious significance.

Synonymous mutations are associated with disease, such as rs3804099 and rs3804100 of *TLR2* [16]. We found that rs3804099 is protective against gastric cancer which is consistent with Wang et al. [47]. As for rs3804100, unfortunately, we only came to the conclusions related to cancer in the subgroup of hospital-based. This conclusion is extremely contingent because of the small number of samples and the limitations of the source of the sample. Taking into account the vast majority of calculations and references, we reserve the conclusion that rs3804100 is not related to cancer. And we are the first meta-analysis involving rs4696480. The overall analysis of B vs. A and BB vs. AA shown that rs4696480 has increased the risk of cancer. At the same time, the calculation results also show that its influence on cancer is particularly obvious among the Caucasian population.

Although our conclusions about -196 to -174del, rs3804099 and rs3804100 are consistent with the previous two meta-analyses, we included more case–control studies, so our meta-analysis is more convincing. And we also clearly observe that ‘ethnic’ factors are critical in assessing the role of *TLR2* in cancer risk. The calculation of -196 to -174del and rs4696480 both found that Caucasians make a significant increase in the cancer risk. And in the model of BB vs. AA and BA vs. AA, rs3804099 deduce the cancer risk in Asians. Furthermore, as the results showing -196 to -174del and rs4696480 are associated with the tumorigenesis, so that these polymorphisms could be a potential biomarker to remind people with the polymorphism pay more attention to the occurrence of cancer, and solve the problem as soon as possible. In the current study, we also evaluated the LD between different polymorphisms of TLR2, we found that there are significantly LD among rs4696480 and rs1898830, rs3804099 and rs3804100. Based on the results, it could guide the researchers to put these polymorphisms together when assess their effect on cancer risks or other bioscience mechanisms. At the same time, we should also be aware of some of the limitations of our article. First of all, based on the results of TSA, we found that the sample size of -196 to -174 del, rs3804100 and rs4696480 is enough to generate the reliable conclusion in the current study, however, larger number of patients are needed to confirm the effect of rs3804099, rs1898830 and rs5743708 to cancer risks. Second, we lack in-depth studies of the effects of environment, lifestyle, bacterial infections and other factors of cancer risk.

## Conclusion

Our meta-analysis suggested that -196 to -174del increased the risk of cancer; rs4696480 increases the risk of cancer in Caucasians; rs3804099 reduced the risk of cancer, especially gastric cancer. While there is no direct evidence showing that rs5743708,3804100 and rs1898830 are related to cancer.

## Supplementary Material

Supplementary Figure S1-S6 and Tables S1-S4

## Abbreviations

CEU: Utah residents with Northern and Western European ancestry from the CEPH collection
CHB: Han Chinese in Beijing, China
CHS: Southern Han Chinese, China
FPRP: false-positive report probability
HWE: Hardy–Weinberg equilibrium
JPT: Japanese in Tokyo, Japan
LD: linkage disequilibrium
NOS: Newcastle–Ottawa Scale
OR: odds ratio
*TLR2*: Toll-like receptor-2
TSA: trial sequential analysis
95% CI: 95% confidence interval
