# Supplementary material for: -196 to -174del, rs4696480, rs3804099 polymorphisms of Toll-like receptor 2 gene impact the susceptibility of cancers: evidence from 37053 subjects
Source: Biosci Rep. 2019 Dec 6;39(12):BSR20191698. doi: 10.1042/BSR20191698 (PMC6900473; doi:10.1042/BSR20191698)
Supplement: Supplementary Figure S1-S6 and Tables S1-S4 [file BSR-2019-1698_supp.pdf]

Supplementary table 1. Methodological quality of the included studies according to the Newcastle-Ottawa Scale.

SNP	Author	Year	Adequacy of Case Definition	Representativeness of the Cases	Selection of Controls	Definition of Controls	Comparability Cases/Controls	Ascertainment of Exposure	Same Method of Ascertainment	Non-response rate
(-196 to -174del)	Tahara *et al.*	2007	*	*	*	*		*	*	NA
(-196 to -174del)	Pandey *et al.*	2009	*	*	*	*	*	*	*	NA
(-196 to -174del)	Hishida *et al.*	2010	*	*			*	*	*	NA
(-196 to -174del)	Srivastava *et al.*	2010	*	*	*	*	*	*	*	NA
(-196 to -174del)	Nischalk *et al.*	2011	*		*	*		*	*	NA
(-196 to -174del)	Oliveira *et al.*	2012	*	*	*	*		*	*	NA
(-196 to -174del)	Mandal *et al.*	2012	*	*	*	*	*	*	*	NA
(-196 to -174del)	Theodoropoulos *et al.*	2012	*		*	*	*	*	*	NA
(-196 to -174del)	Singh *et al.*	2013	*	*	*	*	*	*	*	NA
(-196 to -174del)	Bi *et al.*	2014	*	*	*	*		*	*	NA
(-196 to -174del)	Castano-Rodriguez *et al.*	2014	*	*			*	*	*	NA
(-196 to -174del)	Zidi *et al.*	2014	*	*	*	*		*	*	NA
(-196 to -174del)	Devi *et al.*	2015	*	*	*	*	*	*	*	NA
(-196 to -174del)	Proença *et al.*	2015	*	*	*	*		*	*	NA
(-196 to -174del)	Zidi *et al.*	2015	*	*	*	*	*	*	*	NA
(-196 to -174del)	AL-Harras *et al.*	2016	*	*	*	*		*	*	NA
(-196 to -174del)	Huang *et al.*	2018	*	*	*	*	*	*	*	NA
(-196 to -174del)	Messaritakis *et al.*	2018	*	*	*	*		*	*	NA
(-196 to -174del)	Zeng *et al.*	2011a	*	*			**	*	*	NA
rs3804099	Etokebe *et al.*	2009	*	*	*	*		*	*	NA
rs3804099	Zeng *et al.*	2011b	*	*			**	*	*	NA
rs3804099	Xie *et al.*	2012	*	*			*	*	*	NA
rs3804099	Slattery *et al.*	2012	*	*	*	*	*	*	*	NA
rs3804099	Slattery *et al.*	2012	*	*	*	*	*	*	*	NA
rs3804099	Miedema *et al.*	2012	*	*	*	*		**	*	NA
rs3804099	Zeljic *et al.*	2013	*	*	*	*	**	*	*	NA
rs3804099	Semlali *et al.*	2017	*	*	*	*	*	*	*	NA
rs3804099	Semlali *et al.*	2018	*	*	*	*	*	*	*	NA

rs3804099	Tongtawee *et al.*	2018	*	*			*	*	*	NA
rs3804100	Purdu *et al.*	2008	*		*	*	*		*	NA
rs3804100	Etokebe *et al.*	2009	*	*	*	*		*	*	NA
rs3804100	Xie *et al.*	2012	*	*			*	*	*	NA
rs3804100	Miedema *et al.*	2012	*	*	*	*		**	*	NA
rs3804100	Castaño-Rodríguez *et al.*	2014	*	*			*	*	*	NA
rs3804100	Semlali *et al.*	2017	*	*	*	*	*	*	*	NA
rs3804100	Semlali *et al.*	2018	*	*	*	*	*	*	*	NA
rs3804100	Tongtawee *et al.*	2018	*	*			*	*	*	NA
rs4696480	Miedema *et al.*	2012	*	*	*	*		**	*	NA
rs4696480	Gallo *et al.*	2017	*	*	*	*		*	*	NA
rs4696480	Semlali *et al.*	2017	*	*	*	*	*	*	*	NA
rs4696480	Semlali *et al.*	2018	*	*	*	*	*	*	*	NA
rs5743708	Nischalk *et al.*	2011	*		*	*		*	*	NA
rs5743708	Slattery *et al.*	2012	*	*	*	*	*	*	*	NA
rs5743708	Slattery *et al.*	2012	*	*	*	*	*	*	*	NA
rs5743708	Kına *et al.*	2018	*	*	*	*	*	*	*	NA
rs1898830	Xie *et al.*	2012	*	*			*	*	*	NA
rs1898830	Slattery *et al.*	2012	*	*	*	*	*	*	*	NA
rs1898830	Slattery *et al.*	2012	*	*	*	*	*	*	*	NA

This table identifies “high” quality choices with a “star”. A study can be awarded a maximum of 1 star for each numbered item within the Selection and Exposure categories. A maximum of 2 stars can be given for Comparability. *, Yes; NA, not applicable. (http://www.ohri.ca/programs/clinical epidemiology/oxford.htm).

Supplementary table 2. Details of the sensitivity analyses for *TLR2* polymorphism and cancer risk.

SNP	Comparison	Study omitted	Estimate (95% Confident Interval)	Effect Model
(-196 to -174del)	B vs. A	Tahara et al. (2007)	1.3842176 (1.0833399-1.7686585)	Random
		Pandey et al. (2009)	1.3852967 (1.0883054-1.7633349)	
		Hishida et al. (2010)	1.4153796 (1.0891982-1.8392425)	
		Srivastava et al. (2010)	1.3988788 (1.0941139-1.7885357)	
		Zeng et al. (2011a)	1.4531407 (1.1535394-1.8305556)	
		Nischalk et al. (2011)	1.3801254 (1.0811518-1.761775)	
		Oliveira et al. (2012)	1.3466114 (1.0639657-1.7043427)	
		Mandal et al. (2012)	1.389533 (1.0895275-1.7721463)	
		Theodoropoulos et al. (2012)	1.2380068 (1.0824547-1.4159125)	
		Singh et al. (2013)	1.4060576 (1.1006653-1.7961845)	
		Bi et al. (2014)	1.4226668 (1.1177661-1.8107371)	
		Castano-Rodriguez et al. (2014)	1.4321136 (1.1263661-1.8208549)	
		Zidi et al. (2014)	1.4113173 (1.1074009-1.798641)	
		Devi et al. (2015)	1.3978628 (1.0823373-1.8053708)	
		Proenca et al. (2015)	1.3872203 (1.0892484-1.7667044)	
		Zidi et al. (2015)	1.4134914 (1.1091279-1.8013773)	
		AL-Harras et al. (2016)	1.4114381 (1.109862-1.7949597)	
		Huang et al. (2018)	1.3877288 (1.0831736-1.7779155)	
		Combined	1.3920354(1.1039533-1.7552939)	
	BB vs. AA	Tahara et al. (2007)	1.6191133 (1.1074116-2.3672569)	Random
		Pandey et al. (2009)	1.6689134 (1.1418774-2.4392042)	
		Hishida et al. (2010)	1.8226418 (1.1751486-2.8268964)	
		Srivastava et al. (2010)	1.7162418 (1.1617243-2.5354433)	
		Zeng et al. (2011a)	1.8692523 (1.3004427-2.6868577)	
		Nischalk et al. (2011)	1.6466268 (1.1200988-2.4206614)	
		Oliveira et al. (2012)	1.6287422 (1.1182092-2.372366)	
		Mandal et al. (2012)	1.7230891 (1.1648878-2.5487742)	
		Theodoropoulos et al. (2012)	1.4391873 (1.0632966-1.9479607)	
		Singh et al. (2013)	1.7411302 (1.1714258-2.5879016)	
		Bi et al. (2014)	1.7911378 (1.2046455-2.6631689)	

	Castano-Rodriguez et al. (2014)	1.7924392 (1.2086339-2.6582394)	
	Zidi et al. (2014)	1.7909669 (1.1981788-2.6770315)	
	Devi et al. (2015)	1.7879089 (1.1870695-2.6928654)	
	Proenca et al. (2015)	1.7214299 (1.1655397-2.5424454)	
	Zidi et al. (2015)	1.8113037 (1.217249-2.6952751)	
	AL-Harras et al. (2016)	1.7418679 (1.1769091-2.5780275)	
	Huang et al. (2018)	1.6676577 (1.1255744-2.4708116)	
	Combined	1.7159047(1.1779598-2.4995155)	
BA vs. AA	Tahara et al. (2007)	1.4379952 (1.1013559-1.8775312)	Random
	Pandey et al. (2009)	1.4099357 (1.0802039-1.8403181)	
	Hishida et al. (2010)	1.4325813 (1.0812083-1.8981441)	
	Srivastava et al. (2010)	1.4125484 (1.0770715-1.8525165)	
	Zeng et al. (2011a)	1.475433 (1.1450467-1.9011475)	
	Nischalk et al. (2011)	1.4035684 (1.0712777-1.8389293)	
	Oliveira et al. (2012)	1.3656155 (1.0514834-1.7735951)	
	Mandal et al. (2012)	1.4033546 (1.0730686-1.8353013)	
	Theodoropoulos et al. (2012)	1.249367 (1.0883496-1.4342062)	
	Singh et al. (2013)	1.4239892 (1.088258-1.8632944)	
	Bi et al. (2014)	1.446191 (1.112655-1.8797095)	
	Castano-Rodriguez et al. (2014)	1.4140229 (1.0890495-1.8359689)	
	Zidi et al. (2014)	1.4204854 (1.0901513-1.8509163)	
	Devi et al. (2015)	1.400021 (1.0571072-1.8541719)	
	Proenca et al. (2015)	1.4025673 (1.0742663-1.8311986)	
	Zidi et al. (2015)	1.404094 (1.0766704-1.8310896)	
	AL-Harras et al. (2016)	1.4375898 (1.1057117-1.8690807)	
	Huang et al. (2018)	1.4095417 (1.0742522-1.8494798)	
	Combined	1.4082073(1.0920892-1.8158295)	
BB+BA vs. AA	Tahara et al. (2007)	1.4584963 (1.0969443-1.9392155)	Random
	Pandey et al. (2009)	1.4463475 (1.0912107-1.9170641)	
	Hishida et al. (2010)	1.4739951 (1.0930413-1.9877213)	
	Srivastava et al. (2010)	1.4542896 (1.0920157-1.936747)	
	Zeng et al. (2011a)	1.5263284 (1.1706467-1.9900784)	
	Nischalk et al. (2011)	1.4389279 (1.0814754-1.9145266)	

		Oliveira et al. (2012)	1.4002185 (1.0623446-1.8455516)	
		Mandal et al. (2012)	1.4452412 (1.0879238-1.919916)	
		Theodoropoulos et al. (2012)	1.2812951 (1.1065357-1.4836549)	
		Singh et al. (2013)	1.4650761 (1.102227-1.9473739)	
		Bi et al. (2014)	1.488794 (1.1273342-1.9661493)	
		Castano-Rodriguez et al. (2014)	1.4664143 (1.1123278-1.9332169)	
		Zidi et al. (2014)	1.4689747 (1.108464-1.9467359)	
		Devi et al. (2015)	1.4457284 (1.0732414-1.9474938)	
		Proenca et al. (2015)	1.444193 (1.0888978-1.9154172)	
		Zidi et al. (2015)	1.4631933 (1.1032976-1.9404868)	
		AL-Harras et al. (2016)	1.4769711 (1.117746-1.9516453)	
		Huang et al. (2018)	1.4438585 (1.083371-1.9242967)	
		Combined	1.4489661(1.1069692-1.8966225)	
	BB vs. BA+ AA	Tahara et al. (2007)	1.4057066 (1.0187438-1.9396546)	Random
		Pandey et al. (2009)	1.4802103 (1.0636408-2.059927)	
		Hishida et al. (2010)	1.6088248 (1.0950637-2.3636229)	
		Srivastava et al. (2010)	1.5170711 (1.0795485-2.1319141)	
		Zeng et al. (2011a)	1.6309656 (1.171002-2.2716002)	
		Nischalk et al. (2011)	1.4562807 (1.0420877-2.0351007)	
		Oliveira et al. (2012)	1.4511319 (1.0456713-2.0138106)	
		Mandal et al. (2012)	1.5216708 (1.0817105-2.1405747)	
		Theodoropoulos et al. (2012)	1.3125212 (0.99935091-1.7238308)	
		Singh et al. (2013)	1.5333456 (1.085434-2.166091)	
		Bi et al. (2014)	1.5650365 (1.1045121-2.2175758)	
		Castano-Rodriguez et al. (2014)	1.6183692 (1.1438998-2.2896402)	
		Zidi et al. (2014)	1.5730888 (1.1077236-2.2339582)	
		Devi et al. (2015)	1.5849385 (1.1106908-2.2616825)	
		Proenca et al. (2015)	1.5190281 (1.0813057-2.1339443)	
		Zidi et al. (2015)	1.5942779 (1.1277677-2.2537639)	
		AL-Harras et al. (2016)	1.5282739 (1.0857635-2.1511326)	
		Huang et al. (2018)	1.4761039 (1.0477762-2.0795305)	
		Combined	1.5166734(1.0916522-2.1071714)	
rs3804099	B vs. A	Etokebe et al. (2009)	0.96889651(0.79311037-1.1836442)	Random

	Xie et al. (2012)	0.92676383(0.7667833-1.1201224)	
	Miedema et al. (2012)	0.95441699(0.77630502-1.1733943)	
	Slattery et al. (2012)	0.96444345(0.76220924-1.2203357)	
	Zeljic et al. (2013)	0.90798628(0.77780855-1.0599512)	
	Semlali et al. (2017)	0.98396611(0.80367553-1.2047018)	
	Semlali et al. (2018)	1.0058529(0.83297974-1.2146034)	
	Tongtawee et al. (2018)	1.0002049(0.82552308-1.2118498)	
	Zeng et al. (2011b)	1.007781(0.83084249-1.2224008)	
	Combined	0.96741525(0.805536-1.1618255)	
BB vs. AA	Etokebe et al. (2009)	0.83248496(0.58359981-1.1875111)	Random
	Xie et al. (2012)	0.83050156(0.57751393-1.1943139)	
	Miedema et al. (2012)	0.79650563(0.55756348-1.1378456)	
	Slattery et al. (2012)	0.82188183(0.54482174-1.2398363)	
	Zeljic et al. (2013)	0.83790469(0.67976439-1.0328348)	
	Semlali et al. (2017)	0.86173928(0.59840006-1.2409668)	
	Semlali et al. (2018)	0.89638388(0.64419699-1.2472955)	
	Tongtawee et al. (2018)	0.87157202(0.60786855-1.2496743)	
	Zeng et al. (2011b)	0.89229125(0.62907434-1.2656432)	
	Combined	0.84045512(0.60901101-1.1598556)	
BA vs. AA	Etokebe et al. (2009)	0.82675534(0.71513367-0.95579952)	FIXED
	Xie et al. (2012)	0.84389544(0.73035383-0.97508842)	
	Miedema et al. (2012)	0.8227998(0.70942289-0.95429611)	
	Slattery et al. (2012)	0.72886878(0.60421723-0.87923628)	
	Zeljic et al. (2013)	0.83129221(0.71871334-0.96150529)	
	Semlali et al. (2017)	0.83012533(0.71732771-0.96066004)	
	Semlali et al. (2018)	0.83351672(0.72058511-0.96414721)	
	Tongtawee et al. (2018)	0.83189094(0.7195071-0.96182865)	
	Zeng et al. (2011b)	0.86057824(0.73464006-1.0081059)	
	Combined	0.8265341(0.71730049-0.95240227)	
BB+BA vs. AA	Etokebe et al. (2009)	0.84955496(0.74180108-0.97296107)	FIXED
	Xie et al. (2012)	0.85593086(0.74777192-0.97973406)	
	Miedema et al. (2012)	0.84141642(0.7326141-0.96637726)	
	Slattery et al. (2012)	0.76597619(0.64349884-0.91176468)	

		Zeljic et al. (2013)	0.83369786(0.72757339-0.9553017)	
		Semlali et al. (2017)	0.856233(0.74684209-0.98164654)	
		Semlali et al. (2018)	0.86472785(0.75451595-0.99103838)	
		Tongtawee et al. (2018)	0.86316466(0.75228989-0.99038053)	
		Zeng et al. (2011b)	0.89538008(0.77273506-1.0374907)	
		Combined	0.84974157(0.74417662-0.97028139)	
	BB vs. BA+ AA	Etokebe et al. (2009)	0.98531634(0.75018847-1.2941391)	Random
		Slattery et al. (2012)	1.0224282(0.73720181-1.4180096)	
		Xie et al. (2012)	0.90305895(0.71781307-1.1361111)	
		Miedema et al. (2012)	0.95622373(0.72741711-1.2570007)	
		Slattery et al. (2012)	0.99512035(0.7201038-1.375169)	
		Zeljic et al. (2013)	0.96521997(0.77944446-1.195274)	
		Semlali et al. (2017)	1.0094388(0.76200932-1.3372103)	
		Semlali et al. (2018)	1.0407708(0.79820967-1.3570417)	
		Tongtawee et al. (2018)	1.0242591(0.77977443-1.3453976)	
		Zeng et al. (2011b)	1.0333945(0.78642559-1.3579214)	
		Combined	0.99117113(0.76757505-1.2799012)	
rs3804100	B vs. A	Purdu et al. (2008)	1.0509363(0.87773889-1.2583095)	FIXED
		Etokebe et al. (2009)	1.0712918(0.94372904-1.216097)	
		Xie et al. (2012)	1.023598(0.8918919-1.174753)	
		Miedema et al. (2012)	1.0755367(0.94661063-1.2220222)	
		Castano-Rodriguez et al. (2014)	1.0826485(0.94917309-1.2348937)	
		Semlali et al. (2017)	1.0897315(0.95792955-1.239668)	
		Semlali et al. (2018)	1.0973544(0.96575099-1.2468916)	
		Tongtawee et al. (2018)	1.098178(0.96446925-1.2504234)	
		Combined	1.0757035(0.94900288-1.2193197)	
	BB vs. AA	Purdu et al. (2008)	0.69224417(0.39371327-1.2171344)	FIXED
		Etokebe et al. (2009)	0.82257968(0.51609141-1.3110803)	
		Xie et al. (2012)	0.78167593(0.444442-1.3747964)	
		Miedema et al. (2012)	0.79560041(0.49527037-1.2780495)	
		Castano-Rodriguez et al. (2014)	0.84008753(0.50456429-1.3987256)	
		Semlali et al. (2017)	0.87610734(0.54209089-1.4159324)	
		Semlali et al. (2018)	0.82225353(0.50898784-1.3283241)	

		Tongtawee et al. (2018)	0.92980087(0.57338297-1.5077701)	
		Combined	0.82257966(0.51609141-1.3110803)	
	BA vs. AA	Purdu et al. (2008)	0.95118934(0.74209517-1.2191983)	FIXED
		Etokebe et al. (2009)	1.0331113(0.88734871-1.202818)	
		Xie et al. (2012)	1.0672268(0.9164629-1.2427924)	
		Miedema et al. (2012)	1.0441124(0.8955912-1.2172638)	
		Castano-Rodriguez et al. (2014)	1.0305344(0.88151097-1.2047508)	
		Semlali et al. (2017)	1.0410093(0.8920272-1.2148736)	
		Semlali et al. (2018)	1.0668006(0.91554779-1.2430412)	
		Tongtawee et al. (2018)	1.0363233(0.88703543-1.2107363)	
		Combined	1.040501(0.89578847-1.2085915)	
	BB+BA vs. AA	Purdu et al. (2008)	0.93218386(0.73226815-1.1866783)	FIXED
		Etokebe et al. (2009)	1.0282626(0.88614511-1.1931726)	
		Xie et al. (2012)	1.0493138(0.90400296-1.2179821)	
		Miedema et al. (2012)	1.0359852(0.8916021-1.2037492)	
		Castano-Rodriguez et al. (2014)	1.0301102(0.88389486-1.2005129)	
		Semlali et al. (2017)	1.0422701(0.8959319-1.2125105)	
		Semlali et al. (2018)	1.061036(0.9134236-1.2325029)	
		Tongtawee et al. (2018)	1.0438291(0.89639461-1.215513)	
		Combined	1.0354481(0.89429472-1.1988809)	
	BB vs. BA+ AA	Purdu et al. (2008)	1.3592938(0.97788465-1.889466)	FIXED
		Etokebe et al. (2009)	1.3427418(0.98680955-1.8270552)	
		Xie et al. (2012)	0.76624233(0.43729138-1.3426455)	
		Miedema et al. (2012)	1.3311722(0.97659153-1.814494)	
		Castano-Rodriguez et al. (2014)	1.4226214(1.0304278-1.9640886)	
		Semlali et al. (2017)	1.4013143(1.0246511-1.9164394)	
		Semlali et al. (2018)	1.3562951(0.99298555-1.852531)	
		Tongtawee et al. (2018)	1.4469726(1.0552281-1.9841487)	
		Combined	1.3427418(0.98680954-1.8270552)	
rs4696480	B vs. A	Miedema et al. (2012)	1.1666657(0.93330896-1.4583688)	FIXED
		Gallo et al. (2017)	1.1498516(0.94730139-1.3957106)	
		Semlali et al. (2017)	1.2602687(1.0255111-1.5487664)	
		Semlali et al. (2018)	1.2888148(1.0570656-1.5713724)	

		Combined	1.2160064(1.0187157-1.4515055)	
	BB vs. AA	Miedema et al. (2012)	1.3694062(0.89078385-2.1051946)	FIXED
		Gallo et al. (2017)	1.3104771(0.89652187-1.9155701)	
		Semlali et al. (2017)	1.5451926(1.0297643-2.3186085)	
		Semlali et al. (2018)	1.6503272(1.115391-2.4418161)	
		Combined	1.4627797(1.0342395-2.0688868)	
	BA vs. AA	Miedema et al. (2012)	1.3224006(0.66976017-2.6109993)	Random
		Gallo et al. (2017)	1.1872793(0.77804178-1.8117695)	
		Semlali et al. (2017)	1.6747332(0.99755496-2.8116057)	
		Semlali et al. (2018)	1.5634577(0.83154303-2.9395955)	
		Combined	1.4065839(0.86725762-2.2813039)	
	BB+BA vs. AA	Miedema et al. (2012)	1.3358485(0.72868836-2.4489083)	Random
		Gallo et al. (2017)	1.2201217(0.84528029-1.7611873)	
		Semlali et al. (2017)	1.6279997(0.9715367-2.7280316)	
		Semlali et al. (2018)	1.5861192(0.92640245-2.7156382)	
		Combined	1.4149311(0.91863568-2.1793514)	
	BB vs. BA+ AA	Miedema et al. (2012)	1.1666807(0.80948448-1.6814948)	FIXED
		Gallo et al. (2017)	1.1461788(0.82732379-1.5879225)	
		Semlali et al. (2017)	1.1172881(0.79788733-1.5645475)	
		Semlali et al. (2018)	1.2501878(0.89752364-1.7414242)	
		Combined	1.1693906(0.87195311-1.5682888)	
rs5743708	B vs. A	Nischalk et al. (2011)	16.521536(11.041318-24.721792)	Random
		Kina et al. (2018)	0.98288077(0.51824826-1.8640767)	
		Combined	4.0756825(0.25461694-65.239915)	
	BA vs. AA	Nischalk et al. (2011)	2.9571428(1.4962763-5.8443046)	Random
		Kina et al. (2018)	0.98214287(0.51079553-1.8884357)	
		Combined	1.6968818(0.57462964-5.0108935)	
	BB+BA vs. AA	Nischalk et al. (2011)	2.2095497(0.50451875-9.6767654)	Random
		Slattery et al. (2012)	2.4511757(0.53151315-11.304071)	
		Slattery et al. (2012)	2.0795665(0.33308411-12.983498)	
		Kina et al.(2018)	1.0068018(0.73271269-1.3834208)	
		Combined	1.8129221(0.57173252-5.7486434)	

rs1898830	B vs. A	Xie et al. (2012)	1.0188407(0.93886125-1.1056333)	FIXED
		Slattery et al. (2012)	0.9835791(0.89551055-1.0803088)	
		Slattery et al. (2012)	1.0013056(0.88363576-1.134645)	
		Combined	1.0030393(0.9276129-1.0845988)	
	BB vs. AA	Xie et al. (2012)	1.0031921(0.83704913-1.2023121)	FIXED
		Slattery et al. (2012)	0.92918319(0.75771797-1.1394496)	
		Slattery et al. (2012)	0.9231993(0.70171112-1.2145981)	
		Combined	0.96050464(0.80895557-1.1404448)	
	BA vs. AA	Xie et al. (2012)	1.0563735(0.94049549-1.1865287)	Random
		Slattery et al. (2012)	0.80378997(0.4504793-1.4342021)	
		Slattery et al. (2012)	0.82878494(0.42668638-1.6098112)	
		Combined	0.97101493(0.76821406-1.2273532)	
	BB+BA vs. AA	Xie et al. (2012)	1.0449898(0.93609422-1.1665533)	Random
		Slattery et al. (2012)	0.82465678(0.4953464-1.3728954)	
		Slattery et al. (2012)	0.84786361(0.46967152-1.5305862)	
		Combined	0.97508254(0.79100631-1.2019954)	
	BB vs. BA+ AA	Xie et al. (2012)	0.97547317(0.82177967-1.1579112)	FIXED
		Slattery et al. (2012)	0.97476375(0.81043571-1.1724118)	
		Slattery et al. (2012)	0.98304194(0.77480137-1.2472506)	
		Combined	0.97685901(0.83481723-1.1430688)	

Supplementary table 3. *P* values of the Egger's test for *TLR2* polymorphism.

Polymorphisms	Subgroup	Egger's test P > |t|
(-196 to -174del)	Overall	0.291
	Asian	0.593
	Caucasian	0.983
	African	0.889
	PB	0.791
	HB	0.598
	Gastric Cancer	0.459
	Breast cancer	0.768
	Cervical cancer	0.14
	Y	0.261
	N	0.108
rs3804099	Overall	0.991
	Asian	0.772
	Caucasian	0.405
	PB	0.939
	HB	0.94
	Y	0.236
	N	0.996
rs3804100	Overall	0.279
	Asian	0.003
	Caucasian	0.578
	PB	0.423
	HB	0.297
	Y	0.312
rs4696480	Overall	0.937

	PB	0.553
	Y	0.029
rs1898830	Overall	0.494

Supplementary table 4. Details of the linkage disequilibrium analysis for disequilibrium analysis for TLR2 polymorphisms in populations from 1000 genomes Phase 3.

L1	L2	D'	LOD	R^2	CI_{low}	CI_{high}	Dist	T-int
CHB+CHS								
rs4696480	rs1898830	0.98	74.79	0.905	0.94	1	1327	105.94
rs4696480	rs3804099	0.945	16.58	0.279	0.83	0.99	17530	-
rs4696480	rs3804100	0.938	14.51	0.241	0.8	0.98	18283	-
rs4696480	rs5743708	1	0.06	0.003	0.04	0.97	19191	-
rs1898830	rs3804099	0.948	18.09	0.298	0.84	0.99	16203	64.48
rs1898830	rs3804100	0.94	15.19	0.257	0.81	0.99	16956	-
rs1898830	rs5743708	1	0.05	0.003	0.04	0.97	17864	-
rs3804099	rs3804100	0.987	58.89	0.853	0.94	1	753	88.94
rs3804099	rs5743708	1	0.24	0.006	0.05	0.97	1661	-
rs3804100	rs5743708	1	0.28	0.007	0.06	0.98	908	0.63
CEU								
rs4696480	rs1898830	1	18.31	0.528	0.9	1	1327	25.77
rs4696480	rs3804099	0.44	3.63	0.152	0.26	0.58	17530	-
rs4696480	rs3804100	1	2.36	0.085	0.43	1	18283	-
rs4696480	rs5743708	1	1.47	0.047	0.25	1	19191	-
rs1898830	rs3804099	1	13.87	0.415	0.88	1	16203	25.14
rs1898830	rs3804100	1	1.24	0.045	0.2	1	16956	-
rs1898830	rs5743708	1	2.57	0.088	0.45	1	17864	-
rs3804099	rs3804100	1	3.35	0.108	0.56	1	753	12.02
rs3804099	rs5743708	1	1.03	0.037	0.16	0.99	1661	-

rs3804100	rs5743708	0.635	0.03	0.002	0.04	0.96	908	5.1
ESN								
rs4696480	rs1898830	1	1.99	0.064	0.36	1	1327	3.42
rs4696480	rs3804099	0.008	0	0	-0.01	0.22	17530	-
rs4696480	rs3804100	1	1.43	0.075	0.24	1	18283	-
rs1898830	rs3804099	1	4.89	0.154	0.67	1	16203	6.4
rs1898830	rs3804100	0.922	0.08	0.004	0.04	0.97	16956	-
rs3804099	rs3804100	1	0.81	0.031	0.12	0.99	753	2.32
JPT								
rs4696480	rs1898830	1	42.41	1	0.97	1	1327	56.01
rs4696480	rs3804099	0.897	7	0.3	0.69	0.97	17530	-
rs4696480	rs3804100	0.939	6.6	0.285	0.72	0.99	18283	-
rs1898830	rs3804099	0.897	7	0.3	0.69	0.97	16203	27.2
rs1898830	rs3804100	0.939	6.6	0.285	0.72	0.99	16956	-
rs3804099	rs3804100	1	29.6	0.864	0.93	1	753	42.8
YRI								
rs4696480	rs1898830	1	1.56	0.047	0.27	1	1327	5.39
rs4696480	rs3804099	0.004	0	0	-0.01	0.19	17530	-
rs4696480	rs3804100	1	3.83	0.117	0.6	1	18283	-
rs1898830	rs3804099	1	3.4	0.122	0.57	1	16203	7.42
rs1898830	rs3804100	1	0.19	0.006	0.05	0.97	16956	-
rs3804099	rs3804100	1	1.75	0.046	0.31	1	753	5.77

The linkage disequilibrium values were calculated using $r2$ and D’ statistic CI (Confidence Interval); LOD: Log odds score.

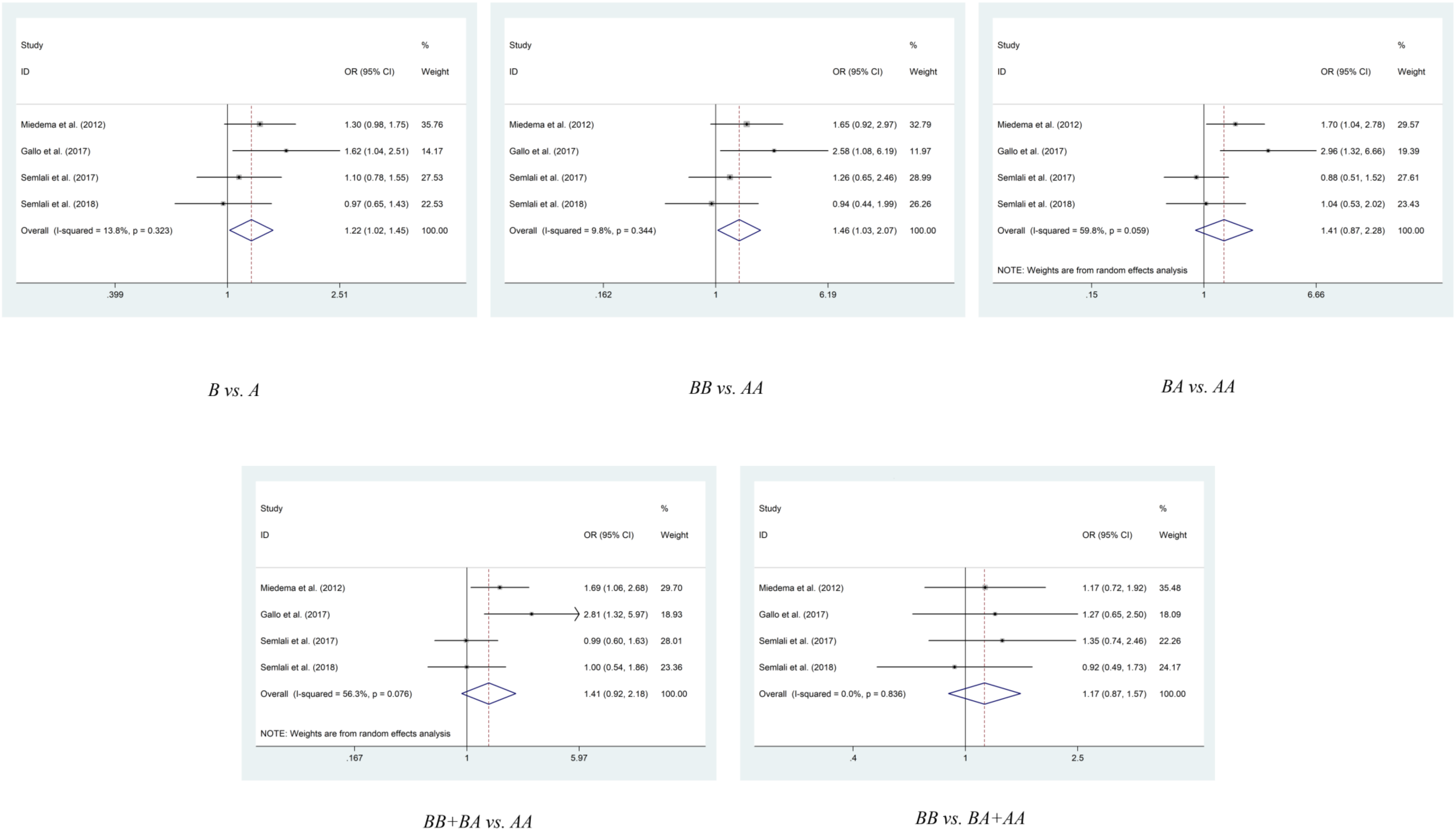

Fig.S1 Meta-analysis of the association between *TLR2 rs4696480* polymorphism and cancer risk.

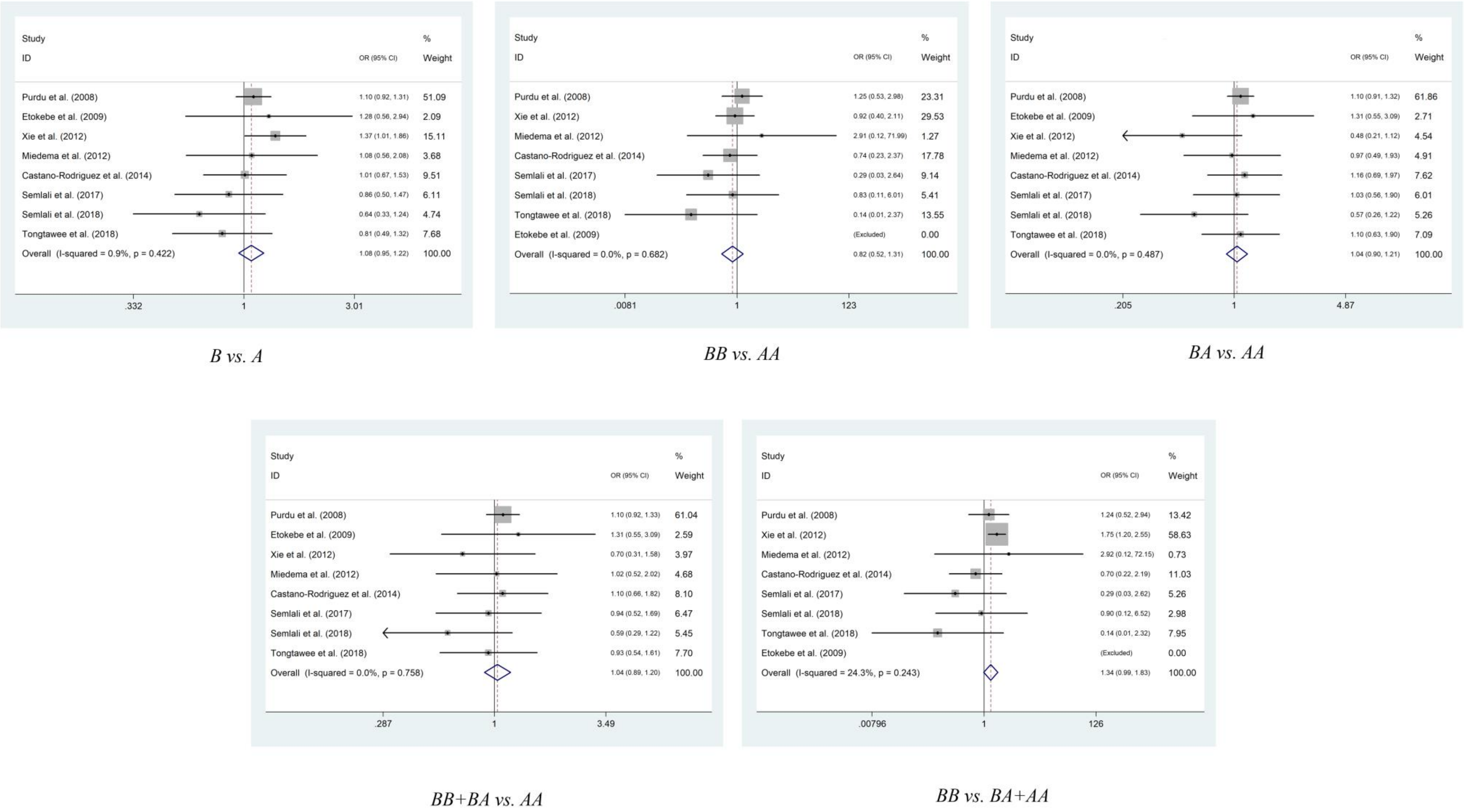

Fig.S2 Meta-analysis of the association between *TLR2 rs3804100* polymorphism and cancer risk.

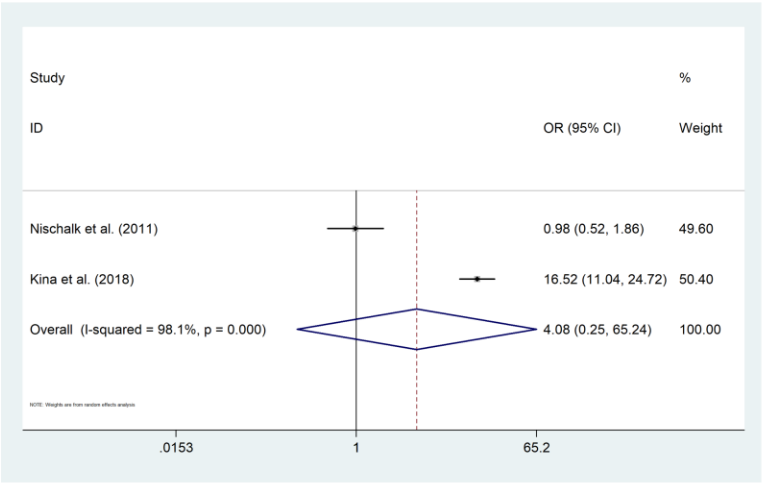

B vs. A

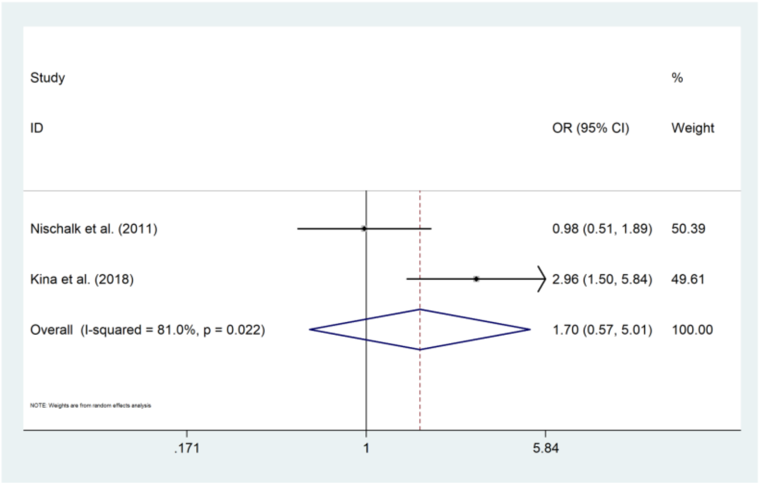

BA vs. AA

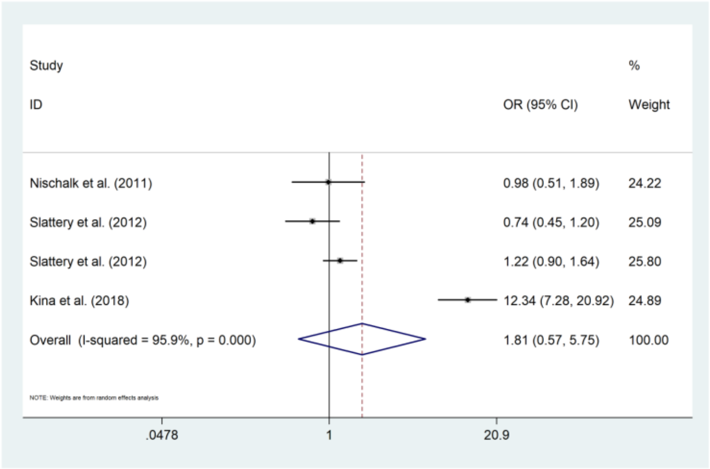

BB+BA vs. AA

Fig.S3 Meta-analysis of the association between *TLR2 rs5743708* polymorphism and cancer risk.

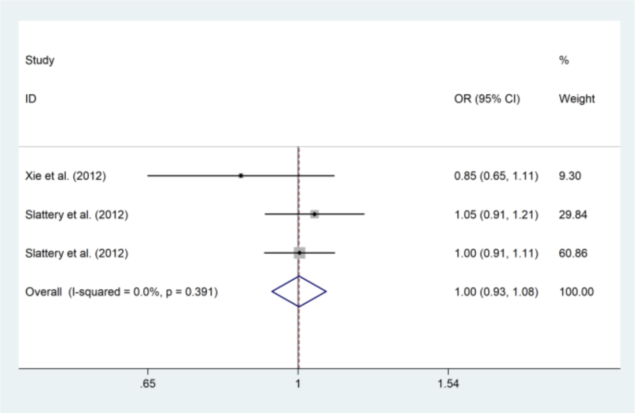

B vs. A

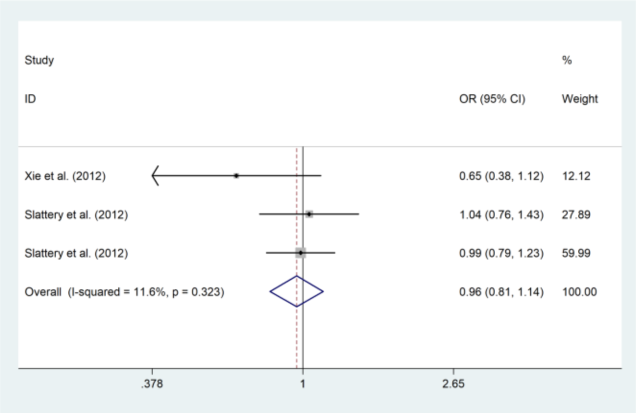

BB vs. AA

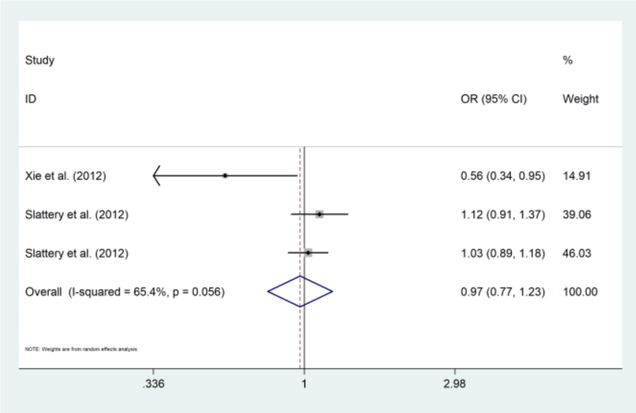

BA vs. AA

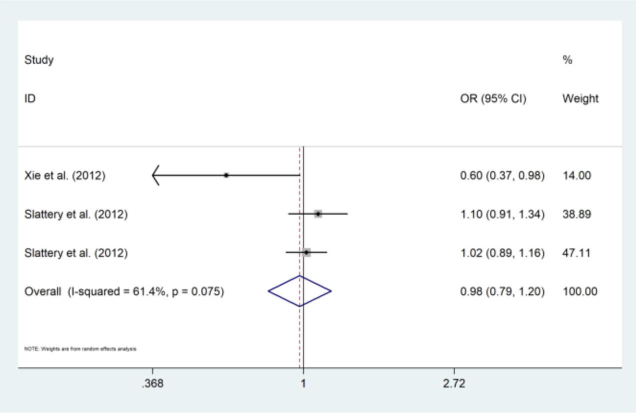

BB+BA vs. AA

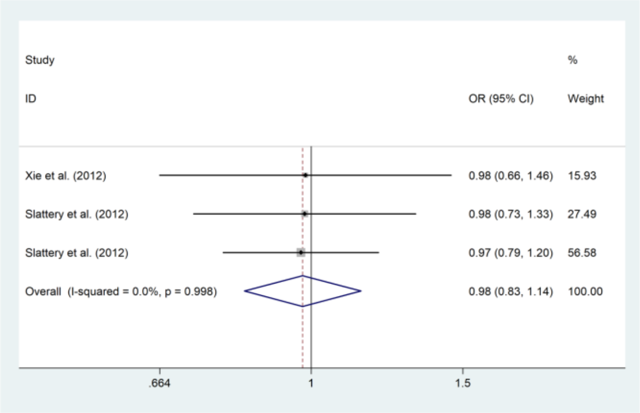

BB vs. BA+AA

Fig.S4 Meta-analysis of the association between *TLR2 rs1898830* polymorphism and cancer risk.

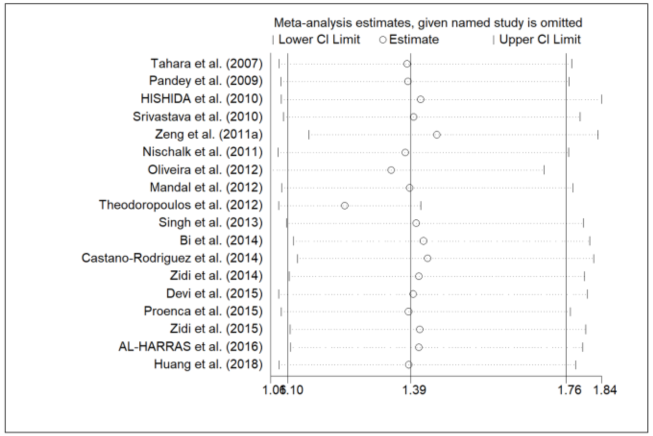

-196 to -174 del

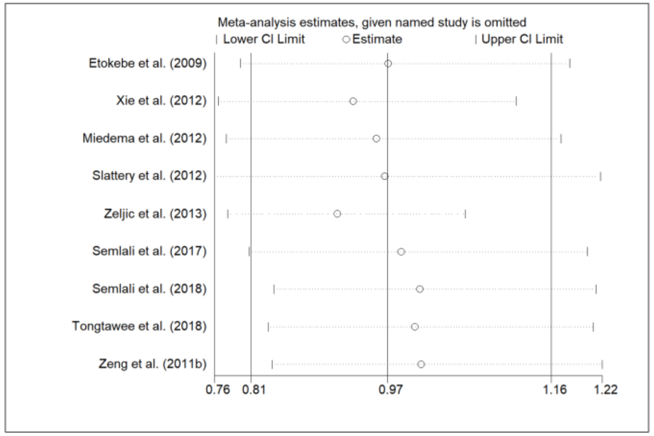

rs3804099

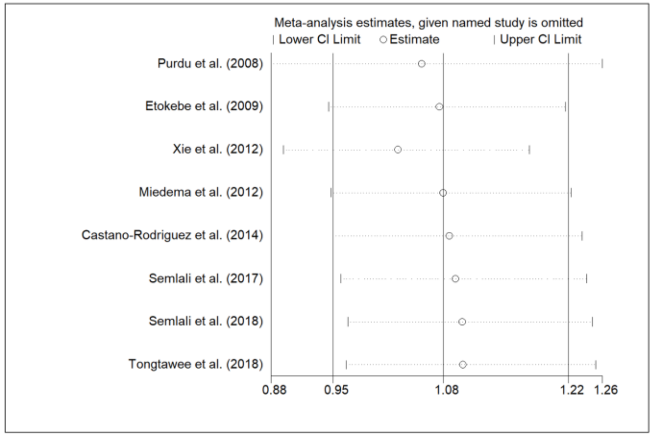

rs3804100

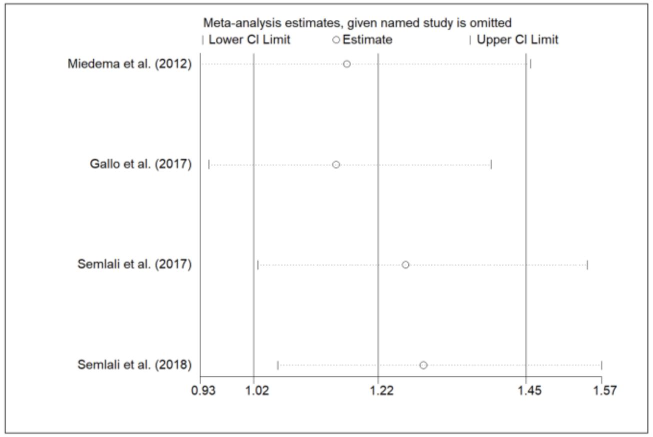

rs4696480

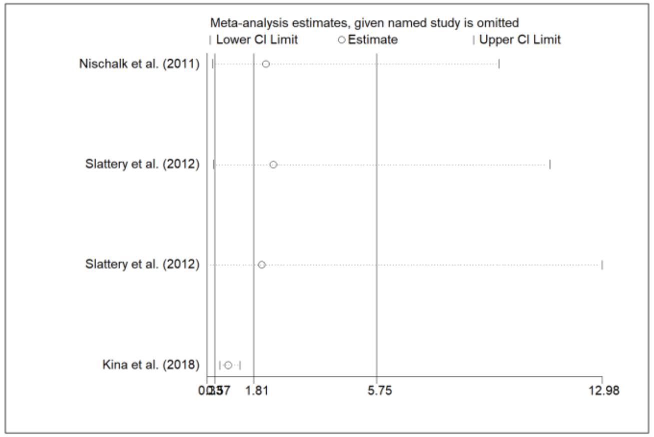

rs5743708

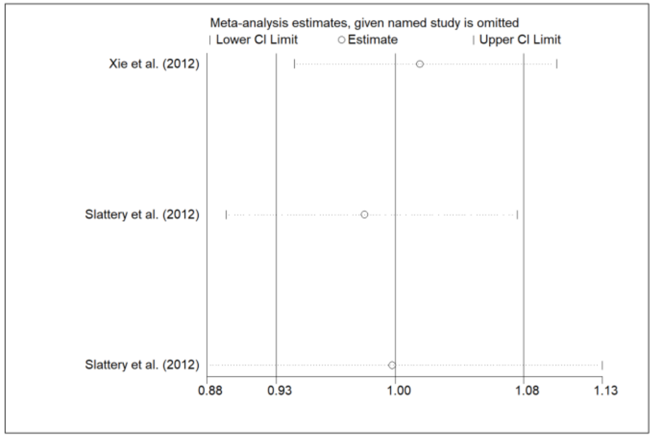

rs1898830

Fig.S5 Sensitivity analysis for TLR2 polymorphism and overall cancer susceptibility (B vs. A, rs5743708, BB+BA vs. AA)

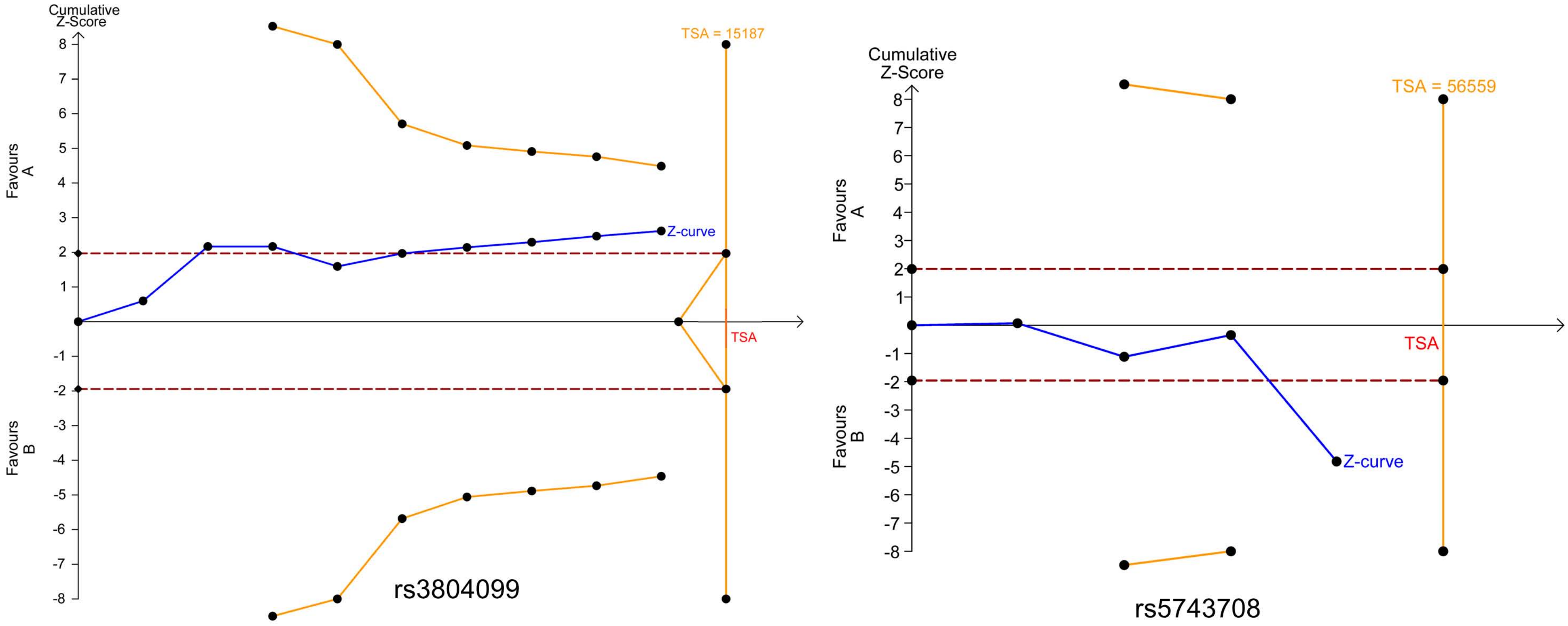

Fig. S6. Trial sequential analysis for *TLR2* polymorphism (rs3804099, rs5743708) under the allele contrast model (B vs. A).
